# PHGDH arginine methylation by PRMT1 promotes serine synthesis and represents a therapeutic vulnerability in hepatocellular carcinoma

**DOI:** 10.1038/s41467-023-36708-5

**Published:** 2023-02-23

**Authors:** Kui Wang, Li Luo, Shuyue Fu, Mao Wang, Zihao Wang, Lixia Dong, Xingyun Wu, Lunzhi Dai, Yong Peng, Guobo Shen, Hai-Ning Chen, Edouard Collins Nice, Xiawei Wei, Canhua Huang

**Affiliations:** 1 West China School of Basic Medical Sciences & Forensic Medicine, and State Key Laboratory of Biotherapy and Cancer Center, West China Hospital, Sichuan University, and Collaborative Innovation Center for Biotherapy, Chengdu, 610041 PR China; 2 Center for Reproductive Medicine, Department of Gynecology and Obstetrics, West China Second University Hospital, Sichuan University, Chengdu, 610041 PR China; 3 Key Laboratory of Birth Defects and Related Diseases of Women and Children (Sichuan University), Ministry of Education, Chengdu, 610041 PR China; 4 Department of Gastrointestinal Surgery, State Key Laboratory of Biotherapy and Cancer Center, West China Hospital, Sichuan University, and Collaborative Innovation Center for Biotherapy, Chengdu, 610041 PR China; 5 Department of Biochemistry and Molecular Biology, Monash University, Clayton, Victoria 3800 Australia; 6 Laboratory of Aging Research and Cancer Drug Target, State Key Laboratory of Biotherapy and Cancer Center, National Clinical Research Center for Geriatrics, West China Hospital, Sichuan University, Chengdu, 610041 PR China

**Keywords:** Cancer metabolism, Methylation, Hepatocellular carcinoma

## Abstract

Serine synthesis is crucial for tumor growth and survival, but its regulatory mechanism in cancer remains elusive. Here, using integrative metabolomics and transcriptomics analyses, we show a heterogeneity between metabolite and transcript profiles. Specifically, the level of serine in hepatocellular carcinoma (HCC) tissues is increased, whereas the expression of phosphoglycerate dehydrogenase (PHGDH), the first rate-limiting enzyme in serine biosynthesis pathway, is markedly downregulated. Interestingly, the increased serine level is obtained by enhanced PHGDH catalytic activity due to protein arginine methyltransferase 1 (PRMT1)-mediated methylation of PHGDH at arginine 236. PRMT1-mediated PHGDH methylation and activation potentiates serine synthesis, ameliorates oxidative stress, and promotes HCC growth in vitro and in vivo. Furthermore, PRMT1-mediated PHGDH methylation correlates with PHGDH hyperactivation and serine accumulation in human HCC tissues, and is predictive of poor prognosis of HCC patients. Notably, blocking PHGDH methylation with a TAT-tagged nonmethylated peptide inhibits serine synthesis and restrains HCC growth in an HCC patient-derived xenograft (PDX) model and subcutaneous HCC cell-derived xenograft model. Overall, our findings reveal a regulatory mechanism of PHGDH activity and serine synthesis, and suggest PHGDH methylation as a potential therapeutic vulnerability in HCC.

## Abstract (web summary)

The role of protein arginine methylation in serine metabolism of cancer cells in hepatocellular carcinoma (HCC) remains to be explored. Here, the authors show that phosphoglycerate dehydrogenase (PHGDH) is activated by PRMT1-mediated R236 methylation, promoting serine synthesis, redox homeostasis and HCC growth.

## Introduction

Cancer cells commonly reprogram metabolic patterns to meet their biomass demands for rapid and sustainable growth^1–3^. Serine metabolism is activated in diverse cancer types to support tumor growth and metastasis, by providing precursors for macromolecule (such as proteins, nucleotides, and lipids) synthesis and once-carbon units for methylation reactions. Moreover, serine synthesis coupling one-carbon metabolism produces glutathione (GSH) and NADPH, two main intrinsic antioxidant agents, to maintain redox balance in cancer cells^4–7^. The intracellular serine pool required for serine metabolism in cancer cells can be replenished by extracellular import or de novo biosynthesis^6,8^. Accordingly, serine metabolism-targeting strategies, including dietary serine/glycine restriction, serine synthesis inhibition, or a combination of each, have been recently explored and exhibit favorable effects for cancer treatment^5,9–13^. However, the underlying mechanism by which serine synthesis is regulated in cancer remains largely unknown.

Phosphoglycerate dehydrogenase (PHGDH) is the first and rate-limiting enzyme in the de novo serine biosynthesis pathway, catalyzing 3-phosphoglycerate (3-PG) to form 3-phosphohydroxypyruvate^14,15^. Recent studies reveal that PHGDH is frequently overexpressed to activate serine synthesis and promote tumor growth in several types of cancer, including breast cancer, non-small-cell lung cancer (NSCLC), and melanoma^16–18^. PHGDH knockdown or inhibitor markedly restrains the growth of these PHGDH-overexpressing cancers, suggesting PHGDH is a promising target for cancer therapy^16–22^. PHGDH upregulation in cancer has been reported to be a consequence of gene amplification or transcriptional upregulation mediated by activating transcription factor 4, nuclear factor erythroid-2-related factor 2, or MYC^18,23–25^. Moreover, the downregulation of E3 ubiquitin ligases (Parkin and RNF5), or upregulation of deubiquitinating enzyme JOSD2, has been reported as crucial mechanism for PHGDH overexpression in breast cancer or lung adenocarcinoma^26–28^. Although the mechanisms underlying PHGDH overexpression in cancer have been well illustrated, the role and regulatory mechanism of PHGDH activity in cancer remains poorly understood.

Protein arginine methylation, a ubiquitous post-translational modification, is catalyzed by protein arginine methyltransferases (PRMTs)^29,30^. Currently, nine PRMTs have been characterized in mammals, which are classified into three different types according to their catalytic specificities. The type I PRMTs (PRMT1-4, PRMT6, and PRMT8) catalyze the formation of mono-methylarginine (me1) and asymmetrical di-methylarginine (me2a), the type II PRMTs (PRMT5 and PRMT9) generate me1 and symmetrical di-methylarginine (me2s), while the type III PRMT (PRMT7) catalyzes the production of me1^30,31^. Emerging evidence has shown that protein arginine methylation plays a crucial role in a wide range of biological processes, such as mRNA splicing, DNA damage, transcription and signal transduction^31,32^. Notably, recent studies demonstrate that PRMTs-mediated protein arginine methylation regulates cancer metabolism, including glucose metabolism, glutamine metabolism, and lipid metabolism^33–37^. However, the role of protein arginine methylation in serine metabolism of cancer cells remains largely unknown.

In this study, we show the essential role of PHGDH methylation for serine synthesis and HCC growth. Although downregulated at mRNA and protein levels, PHGDH is activated by PRMT1-mediated R236 methylation, thereby promoting serine synthesis, redox homeostasis, and HCC growth. Blocking PHGDH methylation by a TAT-tagged nonmethylated peptide inhibits HCC growth with no obvious toxicity, suggesting PHGDH methylation might be a therapeutic vulnerability for HCC treatment.

## Results

### Discordance exists between HCC metabolomic and transcriptomic profiles

**Fig. 1:**
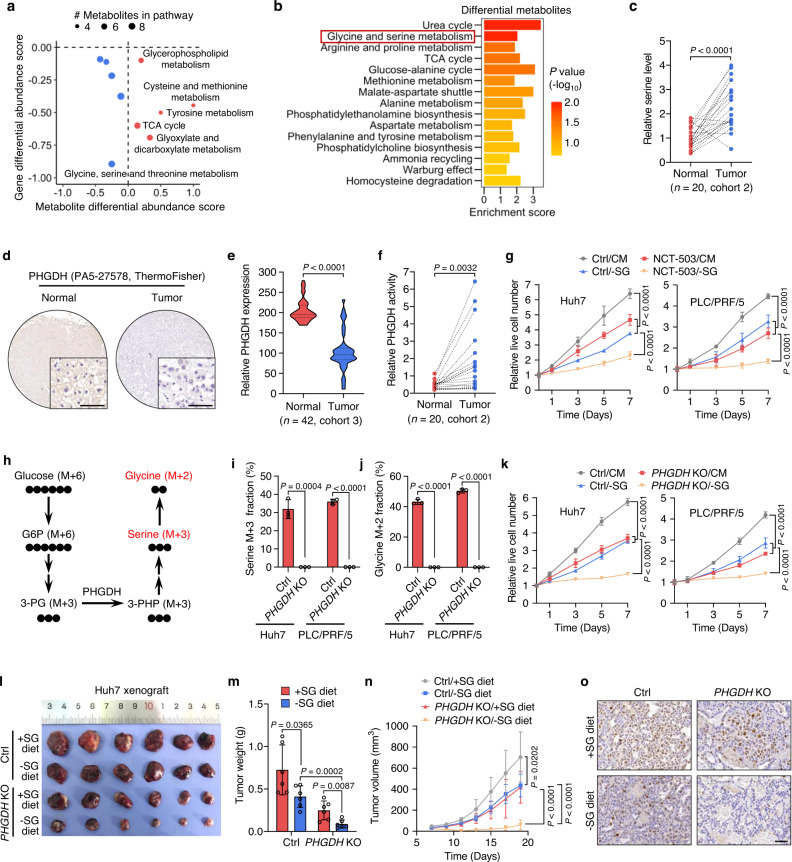
Elevated PHGDH catalytic activity is required for HCC growth. **a** The differential abundance scores of metabolites and genes based on KEGG pathway were plotted against each other. A score of 1 denotes all metabolites in a given pathway increase, while a score of −1 denotes all metabolites in a given pathway decrease. **b** GO analysis of differential metabolites between HCC tissues and normal tissues. Statistical analysis was performed using the two-sided hypergeometric test with Bonferroni correction. **c** Serine levels in human HCC tissues and paired normal tissues (*n* = 20 samples, cohort 2). Statistical analysis was performed using the paired two-tailed Student’s *t*-test. **d**, **e** Representative images (**d**) and quantitative analysis (**e**) of IHC staining using PHGDH antibody (PA5-27578, ThermoFisher) in HCC tissues and paired normal tissues (*n* = 42 samples, cohort 3). Scale bars, 50 μm. Statistical analysis was performed using the paired two-tailed Student’s *t*-test. **f** PHGDH activity in human HCC tissues and paired normal tissues (*n* = 20 samples, cohort 2). Statistical analysis was performed using the paired two-tailed Student’s *t*-test. **g** Growth rates of Huh7 and PLC/PRF/5 cells grown in complete (CM) or serine- and glycine-depleted (-SG) medium treated with or without NCT-503 (20 μM). Data are presented as the mean ± SD (*n*  =  4 independent experiments). Statistical analysis was performed using the two-way ANOVA with Bonferroni correction. **h** Schematic of U-[^13^C]-glucose incorporation into serine and glycine in cells. **i**, **j** Incorporation of U-[^13^C]-glucose carbon into serine (**i**) and glycine (**j**) in parental and *PHGDH* KO cells. Data are presented as the mean ± SD (*n*  =  3 independent experiments). Statistical analysis was performed using the two-tailed Student’s *t*-test. **k** Growth rates of parental and *PHGDH* KO cells grown in CM or -SG medium. Data are presented as the mean ± SD (*n*  =  5 independent experiments). Statistical analysis was performed using the two-way ANOVA with Bonferroni correction. **l–n** Huh7 parental and *PHGDH* KO cells were subcutaneously inoculated into nude mice fed with a control (+SG) or serine- and glycine-free diet (-SG diet). Tumor images (**l**), weight (**m**), and volume (**n**) were presented. Data are presented as the mean ± SD (*n* = 6 mice). Statistical analysis in **m** was performed using the two-tailed Student’s *t*-test, and statistical analysis in **n** was performed using the two-way ANOVA with Bonferroni correction. **o** Representative images of IHC staining for Ki-67 in tumor xenografts. Scale bars, 50 μm. Source data are provided as a Source Data file.

Emerging evidence reveals heterogeneity between metabolomic and transcriptomic profiles in some cancers, such as clear cell renal cell carcinoma (ccRCC)^38^. To investigate the relationship between metabolite and gene expression features in HCC, 29 paired HCC and adjacent normal tissues (cohort 1) were subjected to untargeted metabolomics analysis, and 27 paired tissues from the same cohort were used for RNA-seq analysis (Supplementary Table 1). Unsupervised principal component analysis of both metabolomic and transcriptomic profiles revealed almost completed separation of tumor versus normal samples (Supplementary Fig. 1a, b). A total of 169 metabolites (78 increased and 91 decreased, Supplementary Data 1) and 2798 genes (1605 upregulated and 1193 downregulated) with differential abundance in tumor tissues compared with normal tissues were identified by metabolomics and transcriptomics analyses, respectively (Supplementary Fig. 1c, d). In agreement with previous metabolomics studies^39,40^, we observed decreased levels of succinate, fumarate and malate in tricarboxylic acid cycle (TCA), as well as increased levels of D-phenyllactic acid and L-tryptophan in HCC tissues relative to normal tissues. KEGG pathway enrichment analysis of the differential metabolites revealed alterations in glycine, serine and threonine metabolism, TCA cycle, arginine biosynthesis, as well as alanine, aspartate, and glutamate metabolism (Supplementary Fig. 1e). Besides, enrichment of differential abundance scores was obtained to display the tendency for metabolites in a given pathway to be upregulated or downregulated in HCC tissues compared with normal tissues (Supplementary Fig. 1f)^38^. The differential abundance scores of related metabolic genes were also calculated, and subsequently compared with the metabolite scores. To our surprise, we observed a lack of linear correlation between metabolomics and transcriptomics data. Specifically, five out of ten metabolic pathways showed increased metabolite abundance but reduced gene expression levels (Fig. 1a). These results demonstrate discordance between metabolomic and transcriptomic profiles in HCC.

### Elevated PHGDH catalytic activity is essential for HCC growth

Functional enrichment analysis of differential metabolites revealed two main pathways: urea cycle, and glycine and serine metabolism (Fig. 1b). Given the essential role of the urea cycle in liver for detoxification^41,42^, we had a particular interest in investigating the deregulation of glycine and serine metabolism in HCC development. Among the differential metabolites in this pathway, the level of serine, an oncogenesis-supportive metabolite^4^, was significantly elevated in HCC tissues relative to normal tissues (Supplementary Fig. 2a), which was consistent with previous metabolomics studies^39,43,44^. The increased serine level was further confirmed by serine detection kit in 20 paired HCC tissues and adjacent normal liver tissues (Fig. 1c, cohort 2). We then determined whether the increase in serine level is attributable to the upregulation of PHGDH, the rate-limiting enzyme catalyzing the first committed step of the de novo serine synthesis pathway^4,45^. However, RNA-seq analysis showed a marked decrease in the mRNA level of PHGDH in HCC tissues (Supplementary Fig. 2b). In support of this observation, immunohistochemistry (IHC) analysis of 42 paired HCC samples (cohort 3) using two different commercial PHGDH antibodies demonstrated that the PHGDH protein level was profoundly downregulated in HCC tissues compare with normal tissues (Fig. 1d, e, Supplementary Fig. 2c, d). The decreases in both mRNA and protein levels of PHGDH in HCC tissues were consistent with previous transcriptomics and proteomics data^41,46^. To our surprise, we observed an obvious increase in the catalytic activity of PHGDH in 20 HCC tissues relative to their normal counterparts (Fig. 1f). Together, these data suggest that serine synthesis is activated in HCC due to increased PHGDH activity, although the mRNA and protein levels of PHGDH are downregulated.

Given that serine synthesis promotes tumor growth and survival^4,8^, we next determined whether increased PHGDH activity favors the growth of HCC cells. Treatment with PHGDH inhibitor NCT-503 inhibited the growth and proliferation in two HCC cell lines, Huh7 and PLC/PRF/5 (Fig. 1g, Supplementary Fig. 2e, f). Because serine can be imported extracellularly via its transporters or de novo synthesized through the PHGDH-mediated serine synthesis pathway^6,8^, we combined NCT-503 with serine/glycine starvation (-SG) to deplete serine in HCC cells. Interestingly, serine/glycine deprivation alone led to a decrease in cell growth and proliferation, and further sensitized HCC cells to NCT-503 treatment (Fig. 1g, Supplementary Fig. 2e, f). These results suggest that PHGDH activity is essential for HCC growth. To confirm these observations, we knocked out *PHGDH* expression (*PHGDH* KO) in Huh7 and PLC/PRF/5 cells using CRIPSR/Cas9 technology (Supplementary Fig. 2g). Using U-[^13^C]-glucose tracing (Fig. 1h), we found that 30–40% of the total serine pool and 40–50% of the total glycine pool were labeled by U-[^13^C]-glucose at 24 h of tracing in control parental cells, whereas the incorporation of ^13^C into serine and glycine was totally abrogated when *PHGDH* was knocked out (Fig. 1i, j), which was consistent with a previous report^26^. The *PHGDH* KO cells, theoretically with no PHGDH activity, exhibited reduced growth and proliferation compared with parental cells. In addition, the inhibition of growth and proliferation caused by *PHGDH* KO was further exaggerated when cells were grown in -SG medium (Fig. 1k, Supplementary Fig. 2h, i). These findings were further supported by a mouse xenograft model, in which marked decreases in the size, weight, growth rate, and Ki-67 staining intensity of *PHGDH* KO tumors were observed compared with that of parental cells. Notably, the growth and proliferation of *PHGDH* KO tumors were almost completely blocked when the mice were fed with a -SG diet (Fig. 1l, o, Supplementary Fig. 2j). Collectively, these results indicate that the enhanced PHGDH activity is essential for HCC growth, although PHGDH is downregulated at both mRNA and protein levels in HCC.

### Arginine methylation at R236 elevates PHGDH activity by increasing substrate affinity

**Fig. 2:**
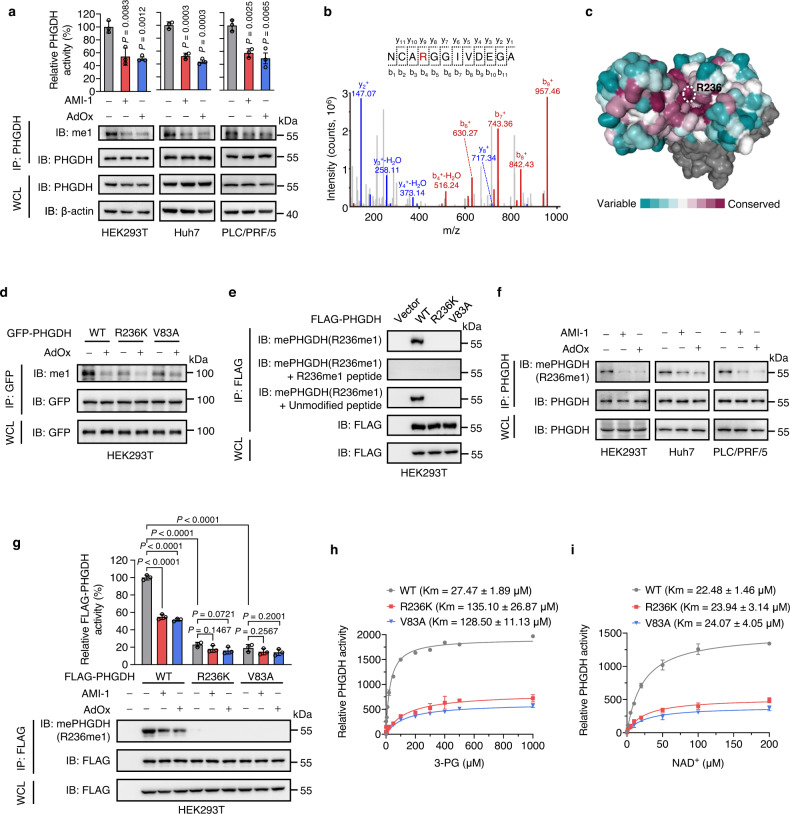
Arginine methylation at R236 elevates PHGDH catalytic activity by increasing substrate affinity. **a** Endogenous PHGDH was immunoprecipitated in cells treated with AMI-1 or AdOx for 24 h. The mono-methylation (me1) level of PHGDH was determined by immunoblotting. PHGDH activity was measured and normalized to PHGDH protein. Data are presented as the mean ± SD (*n*  =  3 independent experiments). Statistical analysis was performed using the two-tailed Student’s *t*-test. **b** MS identification of R236 mono-methylation of FLAG-PHGDH immunopurified by FLAG beads. **c** Sequence conservation of PHGDH protein using the Consurf coloring scheme. Maroon and cyan indicate high and low conservation, respectively. **d** HEK293T cells expressing GFP-PHGDH WT, R236K or V83A were treated with AdOx for 24 h. The mono-methylation (me1) level of immunoprecipitated GFP-PHGDH was determined by immunoblotting. **e** FLAG-PHGDH WT, R236K or V83A was expressed in HEK293T cells. The mono-methylation of immunopurified FLAG-PHGDH was measured using a site-specific antibody against R236 mono-methylation (mePHGDH (R236me1)), which was pre-incubated with the R236 mono-methylated (R236me1) peptide or the unmodified peptide before use. **f** Endogenous PHGDH was immunoprecipitated in cells treated with AMI-1 or AdOx for 24 h. The R236 mono-methylation level of PHGDH was determined by immunoblotting using mePHGDH (R236me1) antibody. **g** HEK293T cells stably expressing FLAG-PHGDH WT, R236K or V83A were treated with AMI-1 or AdOx for 24 h. The R236 mono-methylation level of immunopurified FLAG-PHGDH was determined by immunoblotting using mePHGDH (R236me1) antibody. The activity of immunopurified FLAG-PHGDH was measured and normalized to FLAG-PHGDH protein. Data are presented as the mean ± SD (*n*  =  3 independent experiments). Statistical analysis was performed using the two-tailed Student’s *t*-test. **h**, **i** FLAG-PHGDH WT, R236K or V83A was expressed in HEK293T cells and immunopurified by FLAG beads. The Km value of FLAG-PHGDH for 3-PG (**h**) or NAD^+^ (**i**) was determined. Data are presented as the mean ± SD (*n*  =  3 independent experiments). Source data are provided as a Source Data file.

To investigate the regulatory mechanism of PHGDH activity, we analyzed immunoprecipitated PHGDH from human HEK293T cells by liquid chromatography-tandem mass spectrometry (LC-MS/MS). In addition to CHCHD3 which was reported in another study^27^, the protein arginine methyltransferase PRMT1 was identified with top score as a potential PHGDH-interacting protein (Supplementary Fig. 3a, b). Because the activity of metabolic enzymes can be regulated by post-translational modifications, we, therefore, hypothesized that PHGDH might be methylated by PRMT1, thereby altering its activity. Immunoblotting analysis using antibodies against me1, me2a, and me2s showed that immunoprecipitated GFP-tagged PHGDH indeed underwent mono-methylation and asymmetrical di-methylation (Supplementary Fig. 3c). The mono-methylation of endogenous PHGDH was further confirmed in HEK293T, Huh7, and PLC/PRF/5 cells (Fig. 2a). Of note, the arginine methylation of PHGDH was markedly reduced by PRMT inhibitors, AMI-1 or adenosine dialdehyde (AdOx), with a concomitant decrease in PHGDH activity (Fig. 2a, Supplementary Fig. 3c, d). These data indicate that arginine methylation enhances PHGDH catalytic activity.

The sites of PHGDH arginine methylation were then predicted using the GPS-MSP tool^47^, with R236 showing the highest methylation score (Supplementary Fig. 3e). MS analysis of immunoprecipitated PHGDH confirmed the mono-methylation of PHGDH at R236 (Fig. 2b). Moreover, R236 and its surrounding amino acids (aa) were highly conserved among diverse species (Fig. 2c, Supplementary Fig. 3f), and well matched the consensus RGG/RG methylation motif of PRMT1^32^. To investigate whether R236 is the main methylation site in PHGDH, we mutated R236 to lysine (R236K), which preserved its positive charge but was resistant to PRMTs-mediated methylation. Compared with wild-type (WT), the PHGDH R236K mutant revealed an obvious decreased mono-methylation level. AdOx treatment could attenuate the mono-methylation level of PHGDH WT, but not the R236K mutant (Fig. 2d). These results suggest that R236 is a major methylation site of PHGDH.

To better understand R236 methylation of PHGDH, we generated a site-specific antibody against mono-methylated R236 (mePHGDH (R236me1)), the specificity of which was verified by dot blot and peptide competition analyses (Fig. 2e, Supplementary Fig. 3g). This antibody recognized R236 mono-methylation of both exogenous and endogenous PHGDH, the level of which was markedly attenuated by AMI-1 or AdOx treatment (Fig. 2f, g). Moreover, the R236K mutant showed obvious reduced level of R236 mono-methylation in comparison with WT, accompanied by a significant decrease in PHGDH activity (Fig. 2g). Consistent with our observations in Fig. 2a, AMI-1 or AdOx treatment suppressed the activity of PHGDH WT, but did not alter the activity of R236K mutant (Fig. 2g). Together, these data demonstrate that R236 methylation increases PHGDH activity.

We next asked how arginine methylation enhances PHGDH activity by examining the affinity of PHGDH for its substrate 3-PG and coenzyme NAD^+^. Interestingly, the Km value of PHGDH for 3-PG, but not NAD^+^, was elevated by 2- to 3-fold following AMI-1 or AdOx treatment (Supplementary Fig. 3h, i). In agreement with this observation, R236K mutation resulted in ~5-fold increase in the Km value for 3-PG, whereas the Km value for NAD^+^ remained unchanged (Fig. 2h, i). These results indicate that R236 methylation elevates PHGDH activity by increasing its affinity for the 3-PG substrate.

### PRMT1 methylates PHGDH at R236 and elevates its catalytic activity

**Fig. 3:**
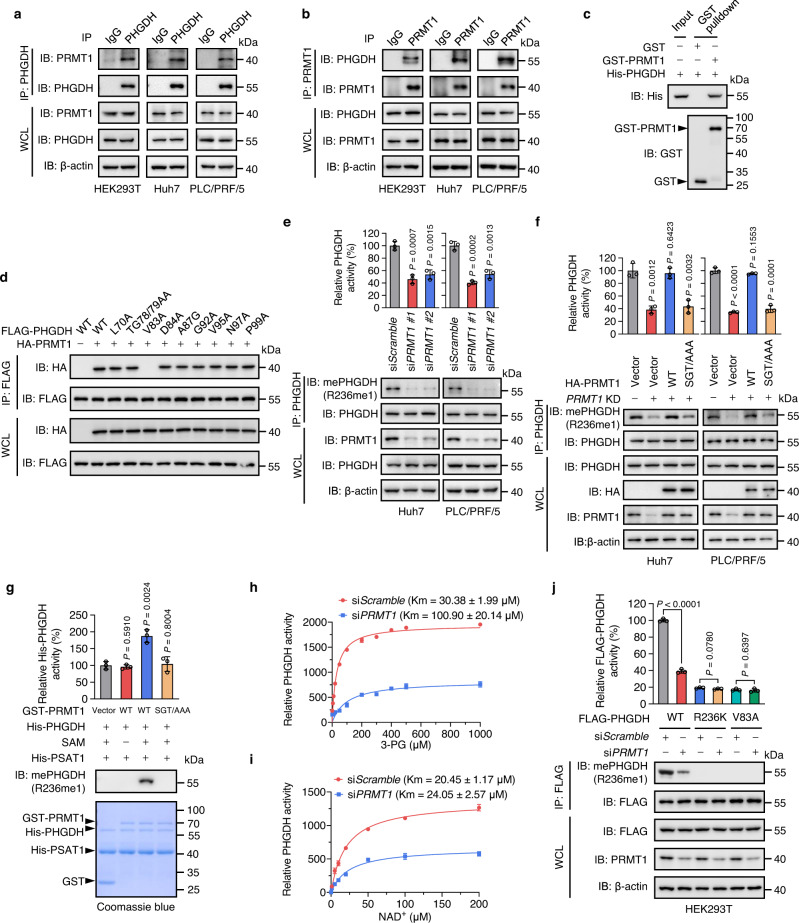
PRMT1 interacts with, methylates, and activates PHGDH. **a, b** Reciprocal co-IP analysis of PHGDH and PRMT1 in HEK293T, Huh7 and PLC/PRF/5 cells. IgG was used as a negative control. **c** Recombinant GST-PRMT1 was incubated with recombinant His-PHGDH, followed by GST pulldown and immunoblotting analysis with GST and His antibodies. **d** FLAG-PHGDH WT or different point mutants were co-expressed with HA-PRMT1 in HEK293T cells. FLAG-PHGDH was immunopurified with FLAG beads, followed by immunoblotting using HA antibody. **e** Endogenous PHGDH was immunoprecipitated in cells transfected with si*Scramble* or si*PRMT1*. Immunoblotting was performed with indicated antibodies. PHGDH activity was measured and normalized to PHGDH protein. Data are presented as the mean ± SD (*n*  =  3 independent experiments). Statistical analysis was performed using the two-tailed Student’s *t* test. **f** Endogenous PHGDH was immunoprecipitated in *PRMT1* KD cells rescued with HA-PRMT1 WT or SGT/AAA mutant. Immunoblotting was performed with indicated antibodies. PHGDH activity was measured and normalized to PHGDH protein. Data are presented as the mean ± SD (*n*  =  3 independent experiments). Statistical analysis was performed using the two-tailed Student’s *t* test. **g** Recombinant GST-PRMT1, His-PHGDH, and His-PSAT1 were incubated with or without S-adenosyl methionine (SAM). The reaction mixture from the in vitro methylation was then subjected to Coomassie Brilliant Blue staining, immunoblotting analysis with mePHGDH (R236me1) antibody, and PHGDH activity assay. Data are presented as the mean ± SD (*n*  =  3 independent experiments). Statistical analysis was performed using the two-tailed Student’s *t*-test. **h**, **i** The Km value of immunoprecipitated PHGDH for 3-PG (**h**) or NAD^+^ (**i**) from cells transfected with si*Scramble* or si*PRMT1*. Data are presented as the mean ± SD (*n*  =  3 independent experiments). **j** HEK293T cells expressing FLAG-PHGDH WT, R236K or V83A were transfected with si*Scramble* or si*PRMT1*, followed by IP with FLAG beads. Immunoblotting was performed with indicated antibodies. The activity of PHGDH was measured and normalized to FLAG-PHGDH protein. Data are presented as the mean ± SD (*n*  =  3 independent experiments). Statistical analysis was performed using the two-tailed Student’s *t*-test. Source data are provided as a Source Data file.

Protein arginine methylation in mammals is catalyzed by protein arginine methyltransferases which comprise nine members, PRMT1-9^30^. To identify the writer of PHGDH R236 methylation, we co-expressed FLAG-PHGDH with different GFP-PRMTs in HEK293T cells and measured their interactions by co-immunoprecipitation (co-IP) assay. PRMT1, PRMT5, and PRMT8 were found to potentially interact with PHGDH (Supplementary Fig. 4a). We selected PRMT1 for further study for the following reasons: (1) LC-MS/MS analysis identified PRMT1 with top score from immunoprecipitated PHGDH (Supplementary Fig. 3a, b); (2) The mono-methylation and asymmetrical di-methylation of PHGDH was consistent with PRMT1’s catalytic properties; (3) The surrounding aa sequence of R236 matched the RGG/RG methylation motif of PRMT1^32^; (4) PRMT5 was recognized as a nonspecific binding protein for FLAG M2 agarose^48^; (5) PRMT8 is mainly expressed in human brain^49,50^. Reciprocal co-IP analysis showed that endogenous PRMT1 interacted with PHGDH in HEK293T, Huh7, and PLC/PRF/5 cells (Fig. 3a, b). To determine whether PRMT1 directly binds PHGDH, GST pulldown assay was conducted using recombinant human GST-PRMT1 and His-PHGDH proteins. Of note, a direct interaction between PRMT1 and PHGDH was observed (Fig. 3c).

We next mapped the domain of PHGDH that binds PRMT1 by co-expressing different truncated GFP-PHGDH with HA-PRMT1 in HEK293T cells for co-IP assay (Supplementary Fig. 4b). The substrate binding domain 1 (SBD1, 1-102aa) of PHGDH was found to be required for PRMT1 binding (Supplementary Fig. 4c). This binding region was further narrowed down to 70-102aa of PHGDH (Supplementary Fig. 4d). We then mutated the amino acids (L70, T78, T79, V83, D84, A87, G92, V95, N97, and P99), which were evolutionally conserved and located on the surface of PHGDH protein in the 70-102aa region (Supplementary Fig. 4e, f). Among them, V83A mutation markedly disrupted the interaction between PHGDH and PRMT1 (Fig. 3d), suggesting that V83 is critical for the binding of PHGDH with PRMT1.

Given that PRMT1 interacts with PHGDH, we determined whether PRMT1 methylates PHGDH, thereby promoting its catalytic activity. siRNA-mediated depletion of PRMT1 significantly attenuated the methylation level of PHGDH in HEK293T, Huh7, and PLC/PRF/5 cells, concomitant with an obvious reduction in the activity of both endogenous and exogenous PHGDH (Fig. 3e, Supplementary Fig. 5a–c). Similar results were observed upon treatment with the PRMT1 inhibitor GSK3368715 (Fig. 5d)^30^. In contrast, PRMT1 overexpression led to significant increased PHGDH methylation and activity (Supplementary Fig. 5e, f). To further validate these findings, we used a short hairpin RNA (shRNA) targeting the 3′UTR of *PRMT1* mRNA to suppress *PRMT1* expression in Huh7 and PLC/PRF/5 cells, and then re-expressed PRMT1 WT or the enzymatically inactive PRMT1 mutant (SGT/AAA)^51^. Reconstituted expression of PRMT1 WT, but not the SGT/AAA mutant, effectively rescued the methylation level and catalytic activity of PHGDH in *PRMT1* knockdown (KD) cells (Fig. 3f). These results suggest that PRMT1 promotes R236 methylation of PHGDH. To investigate whether PRMT1 directly methylates PHGDH, an in vitro methylation assay using recombinant human GST-PRMT1 and His-PHGDH proteins were performed. The R236 methylation of His-PHGDH was detectable in the presence of PRMT1 WT accompanied by an obvious increase of PHGDH activity, whereas incubation of PRMT1 SGT/AAA mutant failed to induce PHGDH methylation and increase PHGDH activity (Fig. 3g). Notably, *PRMT1* KD led to a 3- to 4-fold increase in the Km value of PHGDH for 3-PG, but had no obvious effect on the Km value for NAD^+^ (Fig. 3h, i). Taken together, these data demonstrate that PRMT1 directly binds and methylates PHGDH, resulting in elevated PHGDH catalytic activity by promoting its affinity for 3-PG.

Given our findings that V83 of PHGDH is critical for PRMT1 binding, we presumed that mutation of V83 might reduce PHGDH methylation and inhibit PHGDH activity. Indeed, V83A mutation led to an appreciable decrease in PHGDH methylation and activity, to a similar level observed with R236K mutation (Figs. 2d, e, 2g, 3j). Compared with PHGDH WT, treatment with AMI or AdOx, or *PRMT1* KD resulted in a negligible change in R236 methylation and activity of both R236K and V83A mutants (Figs. 2d, 2g, 3j). Moreover, V83A mutation of PHGDH showed a ~5-fold elevation in the Km value of PHGDH for 3-PG, but not NAD^+^, akin to what we have observed for the R236K mutation (Fig. 2h, i). It has been reported that R236 is located at the catalytic center and is critical for PHGDH activity^52^. To exclude the possibility that R236K mutation blocks PHGDH activity regardless of reduced R236 methylation, we also included the V83A mutant, a methylation-defective surrogate, in the following functional studies.

### PHGDH methylation mediated by PRMT1 promotes serine synthesis and ameliorates oxidative stress

**Fig. 4:**
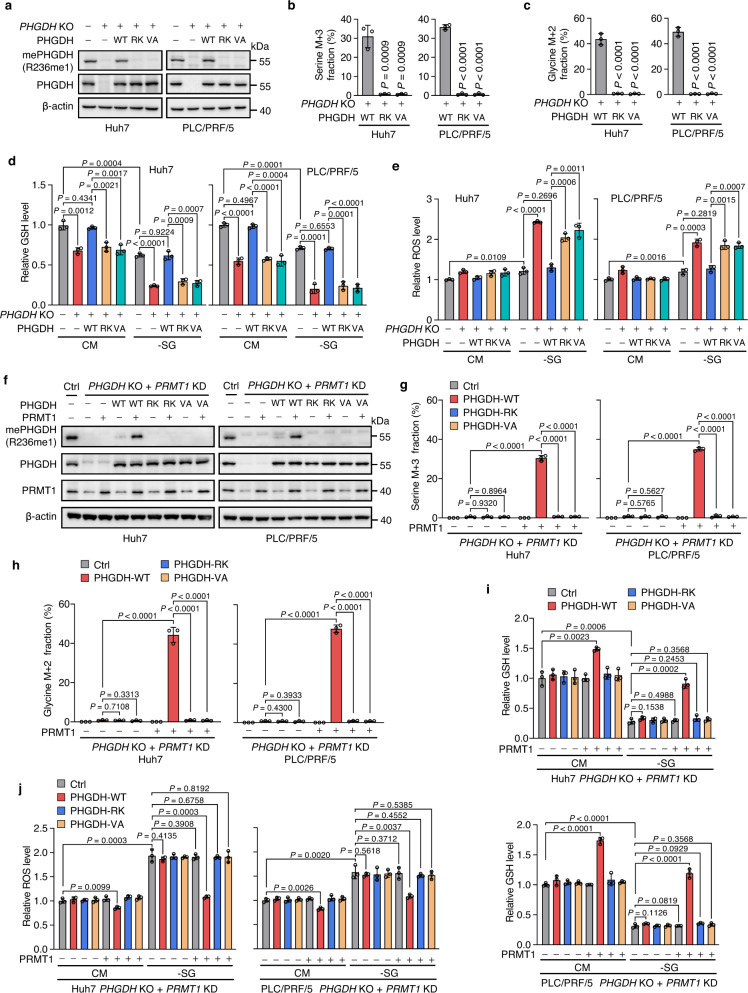
R236 methylation of PHGDH mediated by PRMT1 promotes serine synthesis and ameliorates oxidative stress. **a**–**e** *PHGDH *was knocked out (*PHGDH* KO) using CRIPSR/Cas9 technology, followed by re-expression of PHGDH WT, R236K (RK) or V83A (VA). Immunoblotting was performed with indicated antibodies (**a**). Incorporation of U-[^13^C]-glucose carbon into serine (**b**) and glycine (**c**) was detected by LC-MS/MS. GSH (**d**) and ROS (**e**) levels were measured in these cells grown in CM or -SG medium. Data in **b**–**e** are presented as the mean ± SD (*n*  =  3 independent experiments). Statistical analysis was performed using the two-tailed Student’s *t*-test. **f**–**j** *PHGDH* KO plus *PRMT1* KD cells were rescued with PHGDH WT, R236K (RK) or V83A (VA), combining with or without PRMT1 re-expression. Immunoblotting was performed with indicated antibodies (**f**). Incorporation of U-[^13^C]-glucose carbon into serine (**g**) and glycine (**h**) was detected by LC-MS/MS. GSH (**i**) and ROS (**j**) levels were measured in cells grown in CM or -SG medium. Data in **g**–**j** are presented as the mean ± SD (*n*  =  3 independent experiments). Statistical analysis was performed using the two-tailed Student’s *t* test. Source data are provided as a Source Data file.

Given that PHGDH is the first rate-limiting enzyme for de novo serine synthesis^4,45^, we next sought to assess whether R236 methylation of PHGDH influences serine synthesis. PHGDH WT, R236K, or V83A mutant was re-expressed approximately equal to physiological levels in *PHGDH* KO cells. As expected, R236 mono-methylation was not observed in *PHGDH* KO cells, while re-introducing PHGDH WT, but not R236K or V83A mutant, restored R236 methylation level (Fig. 4a). U-[^13^C]-glucose tracing showed that re-introducing PHGDH WT markedly rescued ^13^C-glucose-derived serine and glycine levels in *PHGDH* KO cells, but not the R236K or V83A mutant (Fig. 4b, c). These observations were recapitulated by detecting serine and glycine levels using serine and glycine detection kits (Supplementary Fig. 6a, b). These data demonstrate that PHGDH methylation promotes serine synthesis in HCC cells.

Serine synthesis supports tumor cell growth by maintaining intracellular redox homeostasis, promoting macromolecule synthesis, and providing once-carbon units for methylation reactions^45^. We and others previously reported that HCC progression is closely associated with oxidative stress, accompanied with a higher antioxidant capacity^53–55^. Therefore, we postulated that the activated serine synthesis by PHGDH methylation might increase the antioxidant capacity, thereby preventing HCC cells from oxidative stress-induced growth inhibition. To test this hypothesis, we examined the levels of GSH and NADPH, which are two major cellular reductants and downstream products of serine synthesis pathway^15^. The GSH level, GSH/GSSG and NADPH/NADP^+^ ratios were profoundly reduced in *PHGDH* KO cells grown in complete medium (CM), which was consistent with a previous study^56^, and were rescued by reconstituted expression of PHGDH WT, but not the R236K or V83A mutant (Fig. 4d, Supplementary Fig. 6c, d). Interestingly, serine/glycine deprivation diminished the GSH level, GSH/GSSG ratio, and NADPH/NADP^+^ ratio in parental cells, and resulted in a further decrease in *PHGDH* KO cells. This decrease was restored by re-expressing PHGDH WT in a manner similar to that of cells grown in CM medium, but not when R236K or V83A mutant was re-expressed (Fig. 4d, Supplementary Fig. 6c, d). The reduction in GSH and NADPH production caused a prominent accumulation of reactive oxygen species (ROS) levels (Fig. 4e). Collectively, these results illustrate that PHGDH methylation prevents ROS accumulation and maintains redox balance by activating serine synthesis in HCC cells.

To determine the role of PRMT1 in PHGDH methylation-mediated stimulation of serine synthesis and elevation of antioxidant capacity, we knocked down *PRMT1* using a shRNA targeting the 3′UTR of *PRMT1* mRNA in *PHGDH* KO cells, followed by re-expression of PHGDH WT, R236K or V83A mutant in combination with or without PRMT1 re-expression. R236 methylation of PHGDH was only observed by re-expressing both PHGDH WT and PRMT1 in *PHGDH* KO plus *PRMT1* KD cells (Fig. 4f). In the absence of PRMT1 expression, rescuing with PHGDH WT, R236K, or V83A mutant failed to restore the reduced serine and glycine levels resulted from *PHGDH* KO. A combination of re-expressing PRMT1 and PHGDH WT, but not its R236K or V83A mutant, appreciably restored serine and glycine levels in *PHGDH* KO plus *PRMT1* KD cells (Fig. 4g, h, Supplementary Fig. 6e, f), suggesting PRMT1 promotes serine synthesis by methylating PHGDH at R236. Of note, the GSH level, GSH/GSSG ratio, and NADPH/NADP^+^ ratio in *PHGDH* KO plus *PRMT1* KD cells could be elevated only when PHGDH WT and PRMT1 were simultaneously re-introduced (Fig. 4i, Supplementary Fig. 6g, h). In support of this observation, the rescue of *PHGDH* KO-induced ROS accumulation was observed by PHGDH WT re-expression in the presence, but not the absence, of PRMT1. Re-expressing R236K or V83A mutant has negligible effect on the increased ROS levels regardless of the presence or absence of PRMT1 (Fig. 4J). Together, these results demonstrate that PRMT1-mediated PHGDH R236 methylation promotes serine synthesis and ameliorates oxidative stress in HCC.

### PHGDH methylation mediated by PRMT1 promotes HCC growth

**Fig. 5:**
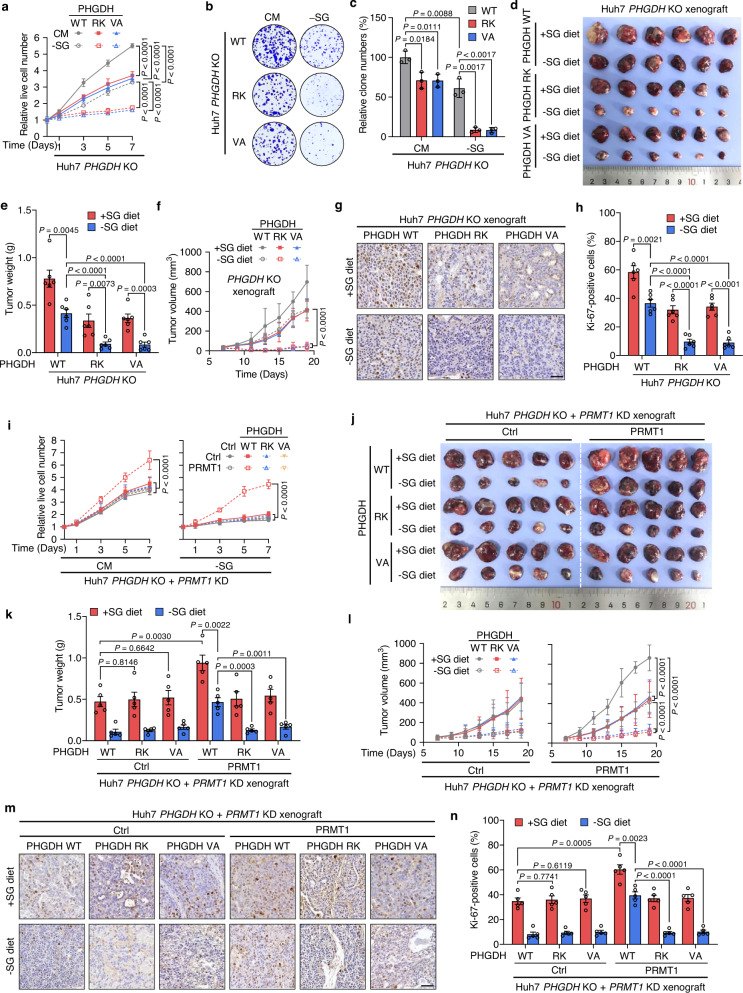
R236 methylation of PHGDH mediated by PRMT1 promotes the growth of HCC cells. **a**–**c** *PHGDH* KO Huh7 cells were re-expressed with PHGDH WT, R236K (RK), or V83A (VA). Growth rates (**a**), colony formation assay (**b**), and quantification of clone numbers (**c**) of these cells grown in CM or -SG medium. Data in **a** are presented as the mean ± SD (*n*  =  5 independent experiments), and statistical analysis was performed using the two-way ANOVA with Bonferroni correction. Data in **c** are presented as the mean ± SD (*n*  =  3 independent experiments), and statistical analysis was performed using the two-tailed Student’s *t*-test. **d**–**f** Cells in **a**–**c** were subcutaneously inoculated into nude mice fed with a +SG or -SG diet. Tumor images (**d**), weight (**e**), and volume (**f**) were shown. Data are presented as the mean ± SD (*n* = 6 mice). Statistical analysis in **d** was performed using the two-tailed Student’s *t*-test, and statistical analysis in **f** was performed using the two-way ANOVA with Bonferroni correction. **g**, **h** Representative images (**g**) and quantitative analysis (**h**) of IHC staining for Ki-67 in tumor xenografts in **d**–**f**. Scale bars, 50 μm. Data in **h** are presented as the mean ± SD (*n* = 6 mice). Statistical analysis was performed using the two-tailed Student’s *t* test. **i** *PHGDH* KO plus *PRMT1* KD Huh7 cells were rescued with PHGDH WT, R236K (RK) or V83A (VA), combining with or without PRMT1 re-expression. Growth rates of these cells grown in CM or -SG medium. Data are presented as the mean ± SD (*n*  =  5 independent experiments), and statistical analysis was performed using the two-way ANOVA with Bonferroni correction. **j**–**l** Cells in **i** were subcutaneously inoculated into nude mice fed with a +SG or -SG diet. Tumor images (**j**), weight (**k**), and volume (**l**) were presented. Data are presented as the mean ± SD (*n* = 5 mice). Statistical analysis in **k** was performed using the two-tailed Student’s *t*-test, and statistical analysis in **l** was performed using the two-way ANOVA with Bonferroni correction. **m**, **n** Representative images (**m**) and quantitative analysis (**n**) of IHC staining for Ki-67 in tumor xenografts in **j–l**. Scale bars, 50 μm. Data in **n** are presented as the mean ± SD (*n* = 5 mice). Statistical analysis was performed using the two-tailed Student’s *t* test. Source data are provided as a Source Data file.

We next investigated whether R236 methylation of PHGDH plays a role in regulating HCC cell growth. The reduction of cell growth and proliferation, resulting from *PHGDH* KO observed in Fig. 1k and Supplementary Fig. 2h, i, was greatly restored by re-expressing PHGDH WT in cells grown in either CM or -SG medium. The decreased cell growth remained marginally affected when re-introducing the R236K or V83A mutant of PHGDH (Fig. 5a–c, Supplementary Fig. 7a–c). To confirm these observations, we generated a mouse xenograft model by subcutaneously inoculating *PHGDH* KO Huh7 cells rescued with PHGDH WT, R236K, or V83A mutant into BALB/c nude mice fed with a control (+SG) or -SG diet. By examining the size, weight, and growth rate of tumors, we found that rescued expression of R236K or V83A significantly inhibited the growth of tumors, as compared with rescue of PHGDH WT. It is noteworthy that when the mice were fed with a -SG diet, R236K or V83A re-expression almost completely blocked tumor growth (Fig. 5d–f). Moreover, the growth inhibition of tumors was accompanied by a marked decrease in the proliferation of tumor cells, as reflected by Ki-67 staining (Fig. 5g, h). These data demonstrate that PHGDH methylation at R236 facilitates the growth and proliferation of HCC cells in vitro and in vivo.

We then determined the role of PRMT1 in PHGDH methylation-induced HCC growth by re-expressing PHGDH WT, R236K mutant, V83A mutant, or PRMT1 in *PHGDH* KO plus *PRMT1* KD cells. Interestingly, the impeded growth of Huh7 and PLC/PRF/5 cells caused by *PHGDH* KO plus *PRMT1* KD was significantly restored only when PHGDH WT and PRMT1 were both re-expressed. Compare with cells grown in CM medium, this restoration was more prominent when serine and glycine in the medium were deprived (Fig. 5i, Supplementary Fig. 7d–h). We further verified these results by implanting *PHGDH* KO plus *PRMT1* KD Huh7 cells rescued with PHGDH WT, R236K mutant, V83A mutant, or PRMT1 into nude mice fed with a control or -SG diet. In line with the data obtained in vitro, the size, weight, and growth rate of tumors, as well as the ratio of Ki-67-positive cells in tumors, were substantially elevated by rescued expression of PHGDH WT plus PRMT1, but not by rescuing with PHGDH WT alone, or rescuing with PHGDH mutants plus PRMT1 (Fig. 5j–n). Furthermore, the growth of HCC cells was markedly reduced by the treatment of PRMT1 inhibitor GSK3368715 both in vitro (Supplementary Fig. 7i–k) and in vivo (Supplementary Fig. 7l–n) in response to serine/glycine starvation. Overall, these results indicate that R236 methylation of PHGDH mediated by PRMT1 promotes the growth and proliferation of HCC cell*s* in vitro and in vivo.

### PHGDH is hypermethylated at R236 by PRMT1 in human HCC tissues

**Fig. 6:**
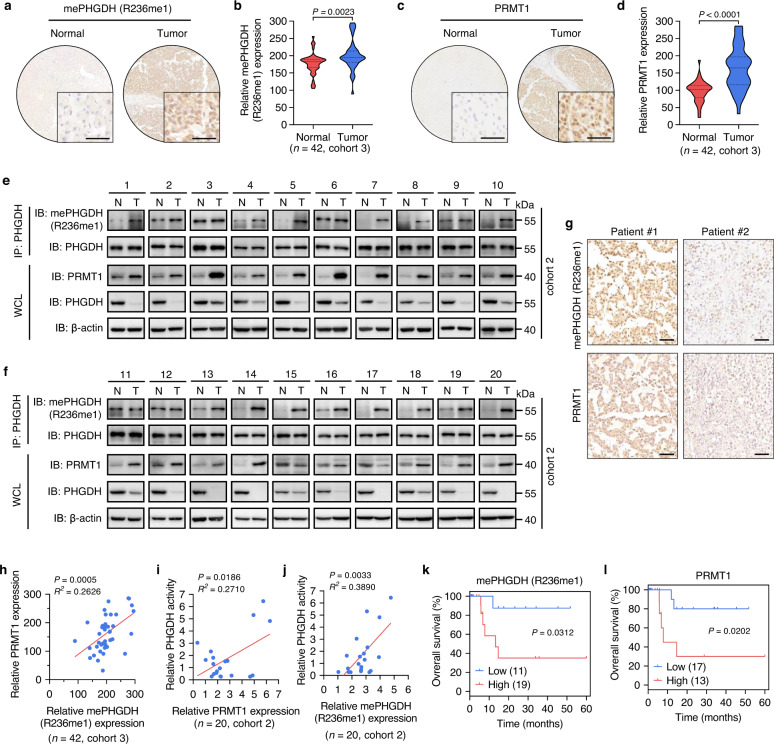
PHGDH is hypermethylated at R236 by PRMT1 in human HCC Tissues. **a**, **b** Representative images (**a**) and quantitative analysis (**b**) of IHC staining using mePHGDH (R236me1) antibody in HCC tissues and paired normal tissues (*n* = 42 samples, cohort 3). Scale bars, 50 μm. Statistical analysis was performed using the paired two-tailed Student’s *t* test. **c**, **d** Representative images (**c**) and quantitative analysis (**d**) of IHC staining for PRMT1 in HCC tissues and paired normal tissues (*n* = 42 samples, cohort 3). Scale bars, 50 μm. Statistical analysis was performed using the paired two-tailed Student’s t-test. **e, f** IP and immunoblotting analysis with indicated antibodies in 20 HCC tissues (T) and paired normal tissues (N). WCL, whole cell lysates. R236 mono-methylation of immunoprecipitated PHGDH was determined and normalized to PHGDH protein (*n* = 20, cohort 2). **g** Representative images of IHC staining with mePHGDH (R236me1) and PRMT1 antibodies in HCC tissues (*n* = 42 samples, cohort 3). Scale bars, 50 μm. **h** Pearson correlation test analyzing the relationship between the IHC staining intensity of mePHGDH (R236me1) and PRMT1 in HCC tissues (*n* = 42 samples, cohort 3). **i** Pearson correlation test analyzing the relationship between PHGDH activity and PRMT1 staining intensity in HCC tissues (*n* = 20 samples, cohort 2). **j** Pearson correlation test analyzing the relationship between PHGDH activity and the staining intensity of mePHGDH (R236me1) in HCC tissues (*n* = 20 samples, cohort 2). **k**, **l** Overall survival of HCC patients based on the IHC staining intensity of mePHGDH (R236me1) (**k**) or PRMT1 (**l**) (*n* = 30 samples). Statistical analysis was performed using the two-sided log-rank test. Source data are provided as a Source Data file.

To evaluate the clinical relevance of PRMT1-mediated PHGDH methylation, IHC analysis was performed on 42 paired HCC samples (cohort 3), the same cohort used for detecting PHGDH protein level (Fig. 1d, e, Supplementary Fig. 2c, d). IHC results revealed that the levels of PHGDH R236 mono-methylation and PRMT1 were both markedly enhanced in HCC tissues relative to adjacent normal liver tissues (Fig. 6a–d). To confirm these observations, we performed co-IP or immunoblotting analysis in 20 paired HCC tissues and adjacent normal liver tissues (cohort 2), the same cohort used for measuring serine level and PHGDH activity (Fig. 1c, f). Consistent with the observations of IHC staining in Fig. 1d, e and Supplementary Fig. 2c, d, PHGDH protein was expressed at lower level in HCC tissues (T) compared with normal tissues (N) (Fig. 6e, f, Supplementary Fig. 8a). Interestingly, HCC tissues displayed an obvious increase in the level of methylated PHGDH compared with normal tissues (Fig. 6e, f, and Supplementary Fig. 8b). The decreased level of PHGDH protein and increased level of PHGDH methylation were also observed in another cohort with 16 paired HCC tissues and adjacent normal liver tissues (cohort 4, Supplementary Fig. 8c–f). In parallel, PRMT1 protein level was found to be upregulated in HCC tissues (cohort 2, Fig. 6e, f, and Supplementary Fig. 8g). Moreover, quantification of the staining intensity showed that PRMT1 protein level in HCC tissues was positively correlated with the amount of methylated PHGDH and PHGDH catalytic activity (Fig. 6g–i, Supplementary Fig. 8h). The R236 methylation level of PHGDH also displayed a positive relationship with PHGDH activity (Fig. 6j). In addition, we compared the overall survival duration of HCC patients, and found that higher levels of both PHGDH methylation and PRMT1 protein were correlated with worse HCC patient prognosis (Fig. 6k, l). Taken together, these results exemplify the requirement for a high level of PRMT1-mediated PHGDH methylation for HCC development.

### Blocking PHGDH methylation with a synthesized peptide suppresses HCC growth

**Fig. 7:**
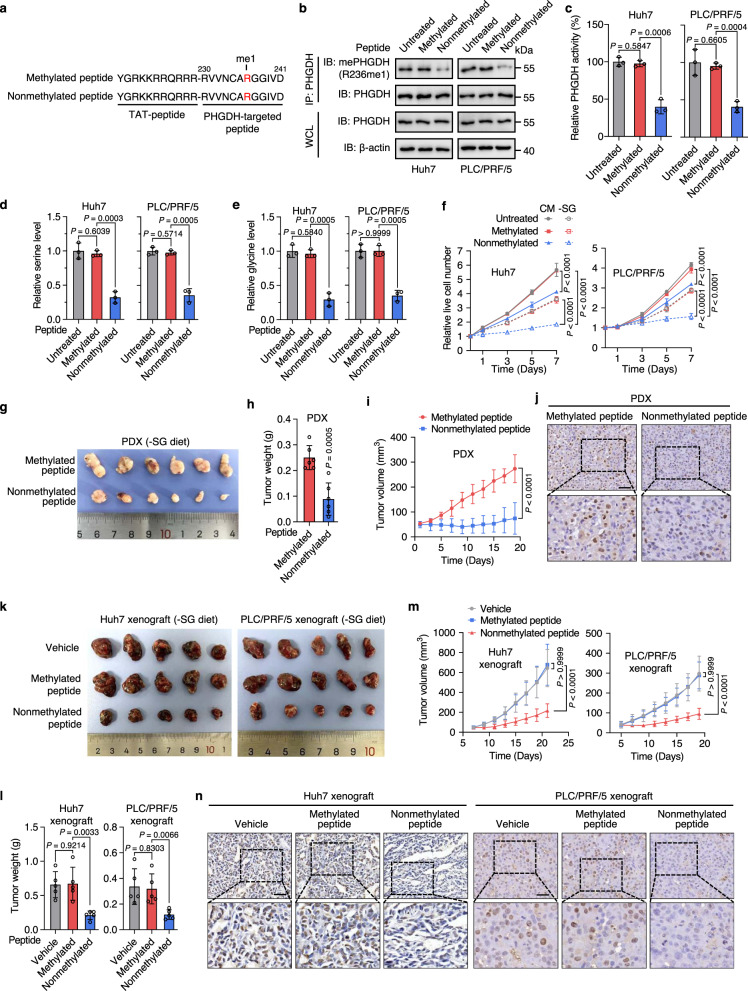
Blocking PHGDH methylation with a synthesized nonmethylated peptide inhibits HCC growth. **a** The aa sequence of the TAT-methylated and TAT-nonmethylated peptides. **b**, **c** Endogenous PHGDH was immunoprecipitated in Huh7 and PLC/PRF/5 cells treated with the methylated or nonmethylated peptides (20 μM) for 24 h. Immunoblotting was performed with indicated antibodies (**b**). PHGDH activity was measured and normalized to PHGDH protein (**c**). Data in **c** are presented as the mean ± SD (*n*  =  3 independent experiments). Statistical analysis was performed using the two-tailed Student’s *t* test. **d**, **e** Total serine (**d**) and glycine (**e**) levels in cells grown in -SG medium with treatment of the methylated or nonmethylated peptides (20 μM) for 24 h. Data are presented as the mean ± SD (*n*  =  3 independent experiments). Statistical analysis was performed using the two-tailed Student’s *t*-test. **f** Growth rates of Huh7 and PLC/PRF/5 cells grown in CM or -SG medium with treatment of the methylated or nonmethylated peptides (20 μM). Data are presented as the mean ± SD (*n*  =  5 independent experiments). Statistical analysis was performed using the two-way ANOVA with Bonferroni correction. **g**–**i** Images (**g**), weight (**h**), and volume (**i**) of HCC PDX tumors from mice fed with a -SG diet and treated with the methylated or nonmethylated peptides. Data are presented as the mean ± SD (*n* = 6 mice). Statistical analysis in **h** was performed using the two-tailed Student’s *t* test, and statistical analysis in **i** was performed using the two-way ANOVA with Bonferroni correction. **j** Representative images of IHC staining for Ki-67 in PDX tumors in **g**–**i**. Scale bars, 50 μm. **k**–**m** Images (**k**), weight (**l**), and volume (**m**) of Huh7 and PLC/PRF/5 tumor xenografts from mice fed with a -SG diet and treated with the methylated or nonmethylated peptides. Data are presented as the mean ± SD (*n* = 5 mice). Statistical analysis in **l** was performed using the two-tailed Student’s *t*-test, and statistical analysis in **m** was performed using the two-way ANOVA with Bonferroni correction. **n** Representative images of IHC staining for Ki-67 in Huh7 and PLC/PRF/5 tumor xenografts in **k**-**m**. Scale bars, 50 μm. Source data are provided as a Source Data file.

Given that PRMT1-mediated PHGDH R236 methylation is necessary for HCC development, we investigated whether PHGDH methylation can be therapeutically targeted. We synthesized a competitive nonmethylated PHGDH peptide containing the R236 methylation site (230-241aa) with a trans-activator of transcription (TAT) peptide tagged at its N-terminal region. A corresponding mono-methylated peptide at R236 and untreated group were used as negative controls (Fig. 7a). Reciprocal co-IP analysis showed that both the methylated and nonmethylated peptides had no obvious effect on the interaction between PHGDH and PRMT1 (Supplementary Fig. 9a–c). In addition, endogenous PRMT1 could be immunoprecipitated with the biotin-conjugated nonmethylated peptide, but not the biotin-conjugated TAT or methylated peptide (Supplementary Fig. 9d). Interestingly, pretreatment with PRMT1 inhibitor GSK3368715 blocked the binding of nonmethylated peptide with PRMT1 (Supplementary Fig. 9e). We then examined the effect of the synthesized peptide on PHGDH methylation and activity. The nonmethylated peptide, but not methylated peptide, markedly inhibited the R236 methylation and enzymatic activity of PHGDH (Fig. 7b, c), concomitant with an obvious decrease in serine and glycine levels (Fig. 7d, e), although the levels of phosphoserine aminotransferase 1 (PSAT1) and phosphoserine phosphatase (PSPH), another two enzymes in serine biosynthesis pathway, were compensatively increased (Supplementary Fig. 9f). These data suggest that the nonmethylated peptide inhibits PHGDH R236 methylation and catalytic activity by binding the active site of PRMT1, but has no obvious effect on the interaction of PHGDH with PRMT1. Moreover, compared with the methylated peptide treatment, cells treated with the nonmethylated peptide displayed repressed GSH level, GSH/GSSG ratio, and NADPH/NADP^+^ ratio, as well as enhanced ROS levels. These alterations were further exacerbated when combining the nonmethylated peptide treatment with serine/glycine depletion (Supplementary Fig. 9g–j). Reconciling with these observations, nonmethylated peptide treatment or serine/glycine deprivation alone inhibited the growth and proliferation of HCC cells, while combined treatment showed a further reduction in HCC cell growth and proliferation (Fig. 7f, Supplementary Fig. 9k, i).

To explore the antitumor efficacy of the nonmethylated peptide in vivo, an HCC patient-derived xenograft (PDX) model was established. The growth of PDX tumors in mice fed with a -SG diet was almost completely prevented by treatment with the nonmethylated peptide, but not the methylated peptide (Fig. 7g–j, Supplementary Fig. 9m). The antitumor effect of the nonmethylated peptide was further confirmed in a subcutaneous mouse xenograft model of Huh7 and PLC/PRF/5 cells (Supplementary Figs. 7k–n, 9n). Importantly, by measuring mice body weight and evaluating the pathological features of major organs, we found treatment with the nonmethylated peptide showed no obvious toxic effects in mice (Supplementary Fig. 10a, b), although a slight loss of weight (<11%) was observed, probably due to dietary serine/glycine restriction as reported previously^10^. Thus, these findings indicate that targeting PHGDH methylation in cooperation with dietary seine/glycine restriction may be a potential therapeutic strategy for HCC treatment.

## Discussion

This study reveals that discordance exists between metabolite abundance and gene expression in HCC. Specifically, the level of serine in HCC is increased, but the expression of PHGDH, a rate-liming enzyme for serine synthesis, is downregulated. The elevated serine level in HCC results from enhanced PHGDH catalytic activity, which is due to PRMT1-mediated arginine methylation of PHGDH at R236. PRMT1-mediated PHGDH methylation and activation promotes serine synthesis and maintains redox homeostasis, supporting HCC growth. Blocking PHGDH methylation using a TAT-tagged nonmethylated peptide combined with dietary serine/glycine restriction synergistically suppresses HCC growth. Overall, our findings provide mechanistic insights into the regulation of PHGDH activity and serine synthesis by PRMT1-mediated arginine methylation, and suggest that inhibition of PHGDH methylation has potential therapeutic applications in HCC.

Metabolic deregulation is a hallmark of cancer. During the past decade, metabolomics, transcriptomics, or integrative analysis of metabolomic and transcriptomic profiles, have been widely conducted to identify potential metabolic biomarkers in HCC^39,41,57–59^. However, these studies mainly focused on the metabolic genes or metabolites with consistent differential abundance between gene expression and metabolite patterns. Here, by combining gene expression and metabolic profile analyses, we find a heterogeneity between metabolomics and transcriptomics data in HCC. Similar discordance has also been observed and reported in other cancer types, such as ccRCC^38^. The heterogeneity between metabolomic profile and gene expression pattern in HCC is not only at the level of metabolic pathways, but also the individual metabolites and metabolic genes. Specifically, we show serine level is significantly elevated in HCC tissues compared with normal tissues, but PHGDH, the rate-limiting enzyme of serine synthesis, is significantly downregulated both at mRNA and protein levels. Therefore, expression of metabolic genes does not always substitute for metabolite abundance. The discordance between metabolomic and transcriptomic profiles may arise from the complex metabolic diversion, post-transcriptional or post-translational regulation of metabolic enzymes, and the presence of enzyme isoforms. In addition, a broader coverage of the metabolome and a larger HCC cohort are needed for future better understanding of such heterogeneity.

Previous studies have reported that PHGDH is frequently overexpressed in a number of different cancer types including breast cancer, NSCLC, and melanoma, due to gene amplification, transcription, or post-translational modification^16–18,23–28^. However, we show an obvious decrease of PHGDH mRNA and protein levels in HCC tissues. Recent transcriptomics and proteomics studies also displayed reduced mRNA and protein levels of PHGDH respectively in HCC tissues^41,46^. Despite the downregulation in mRNA and protein levels, it is worth noting that the catalytic activity of PHGDH is greatly increased in HCC tissues. Indeed, a very early study reported that PHGDH activity was enhanced in tumors from a rat liver cancer model^60^. The increased PHGDH activity enables the stimulation of serine synthesis and resultant serine accumulation to support HCC growth, which is consistent with previous reports showing that PHGDH-mediated serine synthesis is essential for HCC development and sorafenib resistance^23,56,61^. It should be noted that we cannot rule out the contribution of serine transporters to the increased serine level in HCC. Interestingly, a recent study demonstrated that although high PHGDH activity supports cancer cell proliferation, cells with low PHGDH expression exhibit higher metastatic potential in breast cancer^62^. Therefore, the low PHGDH expression may enable HCC metastasis, while the elevated PHGDH activity promotes cell survival and growth. Moreover, in addition to serine, α-ketoglutaric acid (α-KG) is generated as an intermediate, which can replenish TCA cycle and regulate histone demethylation to support cancer cell growth^4,16,17^. Therefore, the role and function of α-KG in HCC is worth further investigation.

We demonstrate that R236 methylation of PHGDH promotes its activity and stimulates serine synthesis. It has been reported that R236 is located in the catalytic center of PHGDH and is responsible for PHGDH activity, likely due to its positive charge property that anchors the carboxyl group of 3-PG^52^. To this end, mutation of arginine to a negatively charged glutamate (R236E), which indeed disrupts the enzyme function of PHGDH, is commonly used as an enzymatically inactive PHGDH mutant^52,63,64^. Interestingly, we show that R236K mutation, which retains its positive charge, also largely suppresses the affinity of PHGDH for 3-PG to abrogate PHGDH activity. Therefore, the positive charge property of R236 does not seem to be a key factor for determining PHGDH activity, thus providing a rationale for the role of R236 methylation in PHGDH activation. This is further supported by using a V83A mutant in parallel in our functional studies, which disrupts the interaction of PHGDH with PRMT1 and exhibits decreased PHGDH methylation level and catalytic activity comparable to that of R236K mutant.

Protein arginine methylation is increasingly associated with cancer development by, at least in part, regulating metabolic reprogramming. For example, PRMT5 mediates R321 methylation of the sterol regulatory element-binding protein and prevents its ubiquitin-proteosome degradation, thereby increasing de novo lipogenesis and accelerating HCC growth^37^. In addition, pyruvate kinase M2 isoform is methylated at R445, R447, and R455 by PRMT4, through which aerobic glycolysis is switched to oxidative phosphorylation to enable the proliferation and metastasis of breast cancer cells^33^. PRMT4 has also been shown to suppress glutamine metabolism in pancreatic ductal adenocarcinoma by methylating malate dehydrogenase 1 (MDH1), or inhibits HCC glycolysis by methylating glyceraldehyde-3-phosphate dehydrogenase, both leading to reduced growth and proliferation of cancer cells^34,35^. However, whether PRMTs are involved in regulating serine metabolism in cancer is unknown. Besides, the role of PRMT1 in metabolic reprogramming of cancer cells remains poorly understood. We demonstrate that PRMT1 promotes serine synthesis in HCC by methylating and subsequently activating PHGDH. This finding reveals a mechanistic link between serine metabolism and protein arginine methylation. PRMT1 has been reported to be upregulated in HCC to promote cell growth and metastasis^65,66^, but the underlying mechanism, especially its direct protein target in HCC, is largely unclear. We show that PRMT1 directly interacts, methylates, and activates PHGDH, thereby promoting serine biosynthesis, maintaining redox homeostasis, and supporting HCC growth.

Dietary serine and glycine limitation has recently attracted much attention for restraining tumor growth both in cultured cancer cells and tumor-bearing mice^9,12,67,68^, highlighting a crucial role of serine metabolism in cancer progression. We found that inhibition of PHGDH activity by either PHGDH inhibitors or blocking PHGDH methylation renders HCC cells more susceptible to serine and glycine deprivation, suggesting both extracellularly imported serine and de novo synthesized serine are crucial for HCC growth. Indeed, it has been reported that depletion of PSAT1 in combination with dietary serine/glycine restriction synergistically suppresses tumorigenesis in c-MYC-driven liver cancer^11^. Different from other studies using PHGDH inhibitors or depleting PHGDH in PHGDH-overexpressing cancers, we surprisingly found that PHGDH inhibition also works for HCC in which PHGDH is downregulated, although the mechanism underlying PHGDH downregulation in HCC is unclear and requires further investigation. This finding supports the notion that PHGDH activity is the key factor in determining the anticancer effect of PHGDH inhibition. Our study thus may broaden the use of PHGDH targeting strategies for cancer therapy, not only in PHGDH-overexpressing cancers, but also in cancer types or subtypes with PHGDH hyperactivation although PHGDH is not overexpressed.

Given the crucial role of PRMT1-mediated PHGDH methylation in HCC growth, we develop a therapeutic peptide to block PHGDH methylation. Through targeting PHGDH methylation, this peptide effectively inhibits serine synthesis and suppresses HCC growth in cooperation with serine and glycine restriction, with no obvious side effects. Together, our study reveals a mechanism by which PHGDH activity and serine metabolism are regulated, and suggests PRMT1-mediated PHGDH methylation as a potential therapeutic target for HCC.

## Methods

### Study approval

All animal studies were performed in accordance with guidelines provided by the Institutional Animal Care and Treatment Committee of Sichuan University. The animals were treated in accordance with relevant institutional and national guidelines and regulations. The maximal allowable tumor size/burden (diameter less than 1.5 cm) was not exceeded. The HCC tissues used in this study were obtained from West China Hospital, Chengdu, with informed written consent from patients. The use of human specimens was approved from the Institutional Ethics Committee of Sichuan University. There was no bias in the selection of patients. 4 different HCC cohorts were used: 29 patients in HCC cohort 1 (14 females and 15 males), 20 patients in HCC cohort 2 (4 females and 16 males), 42 patients in HCC cohort 3 (10 females and 32 males), and 16 patients in HCC cohort 4 (5 females and 11 males). Gender-based analysis revealed that no differences existed in data obtained from HCC patients of different genders (Supplementary Table 2).

### Cell culture

Huh7, PLC/PRF/5 and HEK293T cells were maintained in Dulbecco’s modified Eagle’s medium (DMEM) at 37 °C under 5% CO_2_ supplemented with 10% fetal bovine serum (FBS) and 1% penicillin–streptomycin. All cells were obtained from the Bank of Type Culture Collection of Chinese Academy of Sciences, recently authenticated by fingerprinting of short tandem repeats, and tested negative for mycoplasma. For serine and glycine depletion, cells were cultured in serine- and glycine-free DMEM (US Biological Life Sciences, D9800-03) supplemented with 10% dialyzed FBS.

### Reagents

Antibodies against PHGDH (ab57030, 1:1000 for immunoblotting, and 1:100 for IHC staining), GFP (ab1218, 1:2000; ab32146, 1:5000), PRMT1 (ab190892, 1:1000 for immunoblotting, and 1:100 for IHC staining), HA (ab18181, 1:4000), and Ki-67 (ab16667, 1:500) were purchased from Abcam. Antibodies against PHGDH (PA5-27578, 1:1000 for immunoblotting, and 1:100 for IHC staining) and His (MA1-21315, 1:2000) were purchased from ThermoFisher Scientific. Antibodies against mono-methyl arginine (8015, 1:1000), asymmetric di-methyl arginine (13522, 1:1000), and symmetric di-methyl arginine (13222, 1:1000) were purchased from Cell Signaling Technology. Antibodies against β-actin (AC026, 1:4000), HA (AE036, 1:2000) and FLAG (AE063, 1:2000) were purchased from ABclonal. Antibodies against PSPH (14513-1-AP, 1:1000) and GST (10000-0-AP, 1:2000) were purchased from Proteintech; Antibody against PSAT1 (NBP1-32920, 1:1000) was purchased from Novus Biologicals. The site-specific antibody that recognizes mono-methyl R236 of PHGDH (mePHGDH (R236me1), 1:500 for immunoblotting, and 1:50 for IHC staining) was generated by immunizing rabbits using the synthesized peptide RVVNAA-R(me1)-GGIVDC (GL Biotech, Shanghai).

Periodate oxidized adenosine (AdOx, A7154), 3-phosphoglycerate (3-PG, P8877), NAD^+^ (N1511), glutamate (G1626), S-adenosyl methionine (SAM, A4377), L-serine (S4311), and glycine (G8790) were purchased from MilliporeSigma. AMI-1 (HY-18962), and GSK3368715 (HY-128717A) were purchased from MedChemExpress. NCT-503 (S8619) was purchased from Selleck. U-[^13^C]-glucose (CLM1396) was purchased from Cambridge Isotope Laboratories.

### DNA constructs and mutagenesis

The cDNA encoding full-length human PHGDH was cloned into pcDNA3, pLenti-6.3-puro, pEGFP-N1, or pET28a (+) vector with a 3 × FLAG, HA, GFP, or His tag fusing at the C terminus, or in pQCHIX-hygro with no tag. Polymerase chain reaction (PCR)-amplified human PHGDH cDNA truncates (1-102, 1-266, 1-323, 1-455, 35-533, 69-533, 103-533, 267-533, 324-533, 456-533) were cloned into pcDNA3 vector tagged with FLAG in the C terminus. GFP-PRMTs plasmids were gifts from Dr. Qunying Lei and Dr. Yiping Wang (Fudan University, China). The full-length human PRMT1 was then PCR-amplified and cloned into pcDNA3-HA, pQCHIX-hygro or pGEX-6P-1 vector. The point mutations of PHGDH (R236K, V83A, L70A, TG78/78AA, D84A, A87G, G92A, V95A, N97A, and P99A) and PRMT1 (SGT/AAA) were generated using a Fast Site-directed Mutagenesis Kit (TransGen Biotech, FM111).

### Generation of stable cell pools

To generate *PRMT1* stable knockdown (KD) cell pools, shRNA sequence targeting *PRMT1* (targeting 3′UTR, 5′-TGAGCGTTCCTAGGCGGTTTC-3′) was cloned into pMKO.1-puro vector. The retrovirus was produced by using a two-plasmid packaging system according to the previous report^69^. Cells were infected with the retrovirus and selected with puromycin (2 μg/mL) for 1 week.

To generate *PHGDH* knockout (KO) cells, CRISPR-Cas9 genome editing was performed. sgRNA targeting *PHGDH* (5′-GCTTCTGCCAGACCAATCCA-3′) was cloned into LentiCRISPRv2-puro vector. The lentivirus was produced by using a two-plasmid packaging system according to the previous report^69^. Cells were infected with the lentivirus, and selected with puromycin (2 μg/mL) for 1 week. pQCHIX-hygro vector was used to rescue PHGDH (WT, R236K, or V83A) expression in *PHGDH* KO cells, or PRMT1 (WT or SGT/AAA) expression in *PRMT1* KD cells. The retrovirus was produced using a two-plasmid packaging system and transfected in *PHGDH* knockout or *PRMT1* knockdown cells. Cells were then selected with hygromycin (200 μg/mL) for 1 week.

### Transfection

Huh7, PLC/PRF/5 and HEK293T cells were transfected with various plasmids, packaged viruses or siRNAs using Lipofectamine 3000 (Invitrogen) according to the manufacturer’s protocol. The siRNA oligos (synthesized by GenePharma) were:

si*PRMT1* #1: 5′-CGUCAAAGCCAACAAGUUA-3′

si*PRMT1* #2: 5′-GGACAUGACAUCCAAAGAU-3′

### Immunoblotting and immunoprecipitation

A standard immunoblotting protocol was performed^70^. Briefly, protein lysates of cultured cells or human tissues were prepared with cold RIPA lysis buffer. The protein concentration was quantified using Bradford reagent (Bio-Rad Laboratories). Proteins were then separated by SDS-PAGE, transferred onto PVDF membranes, incubated with antibodies, and visualized by enhanced chemiluminescence reagent (Millipore). For immunoprecipitation, protein lysates were prepared with cold NP-40 buffer (50 mM Tris-HCl (pH 7.4), 150 mM NaCl, 1% NP-40, 1 mM EDTA) containing protease inhibitor cocktail and phosphatase inhibitor cocktail (Bimake). Protein lysates were incubated with FLAG beads (MilliporeSigma, A2220) or streptavidin beads (MilliporeSigma, 69203) at 4 °C for 3 h or primary antibody at 4 °C overnight, followed by incubating with Protein A/G beads (Millipore, IP10) at 4 °C for 3 h. The beads were washed with cold NP-40 buffer, boiled, and analyzed by standard immunoblotting protocols.

### Colony formation assay

Cells were seeded in 24-well plates (600 cells per well). For serine and glycine depletion, cells were washed with PBS at 12 h after cell seeding, then serine- and glycine-replete or depleted medium was added. After 10 days, cells were fixed with 4% paraformaldehyde and stained with crystal violet. Colonies were photographed and counted using Image J software.

### PHGDH activity assay

PHGDH catalytic activity was determined following the previous report^20^. Briefly, FLAG-tagged PHGDH protein was immunopurified with FLAG beads (MilliporeSigma) and then eluted by FLAG peptides (MilliporeSigma). Activity assay was performed in 96-well plates at 28 °C in assay buffer (50 mM Tris-HCl, pH 8.5, 1 mM EDTA, 240 μM 3-PG, 120 μM NAD^+^, and 30 mM glutamate) in a total volume of 100 μL per well. Recombinant human His-PSAT1 protein (80 μg/mL) was added to prevent the product inhibition of PHGDH. PHGDH activity was monitored by measuring the increase of NADH fluorescence (Ex. 340 nm, Em. 460 nm) using a Varioskan LUX Multimode Microplate Reader (ThermoFisher Scientific). To measure the activity of endogenous PHGDH protein in cultured cells or human tissues, PHGDH protein was immunoprecipitated with PHGDH antibody. The beads were suspended in assay buffer and then subjected to fluorescence measurement. To determine the Km value of PHGDH, serial concentrations of 3-PG (0, 1, 5, 10, 20, 50, 100, 200, 300, 400, 500, and 1000 μM) or NAD^+^ (0, 1, 5, 10, 20, 50, 100, and 200 μM) were used to measure PHGDH activity. The Km value was determined using the Michaelis-Menten curve plotted in GraphPad Prism 8.0.

### ROS measurement

To determine the intracellular ROS levels, cells were washed with PBS and loaded with the fluorescent dye 2′,7′-dichlorofluorescein diacetate (H_2_DCF-DA, MilliporeSigma, 35845) at 37 °C for 30 min. The stained cells were washed twice with PBS, trypsinized, and resuspended in PBS. Fluorescent intensity (Ex. 488 nm, Em. 525 nm) was monitored using a Varioskan LUX Multimode Microplate Reader (ThermoFisher Scientific).

### GSH and GSH/GSSG ratio quantification

The intracellular GSH level and GSH/GSSG ratio were determined using GSH and GSSG Assay Kit (Beyotime, S0053) following the manufacturer’s instructions. GSH and GSSG signal intensities were measured at OD of 412 nm using a microplate reader (SpectraMax 190, Molecular devices).

### NADPH/NADP^+^ ratio quantification

The intracellular NADPH/NADP^+^ ratio was determined using the NADP^+^/NADPH Assay Kit (Beyotime, S0179) following the manufacturer’s instructions. NADP^+^ and NADPH signal intensities were measured at OD of 450 nm using a microplate reader (SpectraMax 190, Molecular devices).

### Recombinant protein purification

His-PHGDH, His-PSAT1, GST-PRMT1-WT, or GST-PRMT1-SGT/AAA mutant constructs were transformed into BL21 *E. coli*. Cells were grown at 37 °C to an optical density measure at 600 nm (OD_600_) of ~0.6, and then induced by 0.4 mM IPTG at 18 °C overnight. The cells were harvested and lysed by sonication. Fusion proteins were purified using the Ni^2+^-charged HisTrap Chelating columns (GE Healthcare Life Sciences) or GSTrap HP columns (GE Healthcare Life Sciences). The purity of recombinant proteins was determined by Coomassie Brilliant Blue staining and immunoblotting.

### GST pulldown

GST-PRMT1 fusion protein or GST protein (negative control) was mixed with recombinant human His-PHGDH protein at 4 °C overnight, followed by incubation with GST beads (Smart Lifesciences, SA008005) at 4 °C for 3 h. Beads were then washed with NP-40 buffer, boiled in SDS loading buffer, and subjected to SDS-PAGE and immunoblotting analysis.

### In vitro methylation assay

The in vitro methylation assay was performed following the previous report^71^. Briefly, 5 μg His-PHGDH, 5 μg GST-PRMT1, and 200 μM SAM were mixed in 50 μL reaction buffer (50 mM Tris-HCl, pH 8.0, 20 mM KCl, 5 mM DTT, 4 mM EDTA), and incubated at 37 °C for 1.5 h. The mixture was then boiled with SDS loading buffer, and subjected to SDS-PAGE and immunoblotting analysis.

### Mass spectrometry analysis

The PHGDH-binding proteins in cells were identified by co-IP followed by mass spectrometry (MS) analysis. In brief, HEK293T cells stably expressing FLAG-PHGDH were lysed and subjected to IP using FLAG beads. Immunoprecipitates were separated by SDS-PAGE. The protein bands visualized by Coomassie Brilliant Blue staining were excised and digested in gel with sequencing-grade trypsin (Promega). The digested samples (FLAG-PHGDH-binding proteins) were analyzed on a Q Exactive Plus Orbitrap LC-MS/MS System (ThermoFisher Scientific) and identified using Proteome Discoverer 1.2 software (ThermoFisher Scientific). To identify the potential methylation site for PHGDH, GFP-PHGDH was overexpressed in HEK293T cells and immunoprecipitated with GFP beads, followed by SDS-PAGE and Coomassie Brilliant Blue staining. The GFP-PHGDH protein band was digested in gel by α-lytic protease (MilliporeSigma, A6362), and then analyzed by LC-MS/MS in collaboration with PTM Biolabs (Hangzhou, China).

### RNA-seq analysis

Total RNA from 27 human HCC samples and paired normal liver tissues were extracted and subjected to RNA sequencing with Illumina NovaSeq 6000 by Novogene Inc. (Tianjin, China).

### Metabolomics analysis

Untargeted metabolomic profiling of 29 human HCC tissues and paired normal liver tissues was performed by LC-MS/MS (Agilent 1290 II, Infinity Agilent Technologies; 5600 Triple TOF Plus, AB Sciex) in collaboration with LipidALL Technologies (Changzhou, China). MarkerView (Version 1.3, AB Sciex) and PeakView (Version 2.2, AB Sciex) were used for data processing. Metabolites were identified by comparison with standard references in the HMDB and METLIN databases.

### Differential abundance score

The differential abundance score was calculated as:

Differential abundance score = (No. of increased metabolites − no. of decreased metabolites)/No. of measured metabolites in a given pathway^38^

The differential abundance score can reflect the average and gross alterations for all measured metabolites in a given pathway. A score of 1 denotes all metabolites in a given pathway increase, while a score of −1 denotes all metabolites in a given pathway decrease.

### Serine and glycine measurement using Serine and Glycine Assay Kit

Serine and glycine concentrations in lysates of cells or human tissues were measured using DL-Serine Assay Kit (Biovision, K743) and Glycine Assay Kit (Biovision, K589), respectively, following the manufacturer’s instructions. To determine serine and glycine concentrations in cultured cells, cells were seeded in 6-well plates with serine- and glycine-depleted medium. Fluorescent intensity was measured (Ex. 535 nm, Em. 587 nm) using a Varioskan LUX Multimode Microplate Reader (ThermoFisher Scientific).

### ^13^C-labeled metabolite analysis by LC-MS/MS

5 × 10^6^ cells were cultured in glucose-free medium supplemented with 10% dialyzed FBS and 25 mM U-[^13^C]-glucose for 24 h. Cells were then washed twice with cold PBS and resuspended in cold 80% methanol and 20% ddH_2_O for metabolite extraction. LC-MS/MS analysis of the metabolic abundance was conducted by LipidALL Technologies (Changzhou, China).

### TAT-tagged peptide synthesis

The sequence of synthesized peptide (RVVNCARGGIVD) consisted of 230-241aa of PHGDH containing R236 methylation site, with a cell-penetrating TAT peptide tagged at its N-terminal region to improve cellular uptake. For the methylated peptide, R236 was mono-methylated, whereas R236 remained unmodified for the nonmethylated peptide. For biotin conjugation, the biotin molecule was placed in the N-terminal region of the TAT peptide. All peptides were synthesized, purified to >95% by HPLC, and verified by LC/MS in collaboration with Shanghai Dechi Biosciences Co, Ltd.

### Animal studies

Mice were housed under ambient temperature of 24 ± 2 °C, circulating air, constant humidity of 50 ± 10%, and a 12 h:12 h light/dark cycle. Male mice have been used due to that the gender of the patients has no obvious effect on the observations and conclusions in this study (Supplementary Table 2). In addition, the cell-autonomous effect on xenograft growth was not related with gender. For xenograft studies, 5 × 10^6^ Huh7 or PLC/PRF/5 cells were subcutaneously injected into the flanks of BALB/c nude mice (6-week-old males, HFK Bioscience). For the patient-derived xenograft (PDX) model, tumors from PDX-bearing NSG mice (purchased from IDMO, Beijing) were cut into pieces and subcutaneously implanted into the flanks of NSG mice (6-week-old males). For serine/glycine starvation, mice were placed on a normal diet (HFK Bioscience, 1035) or a serine- and glycine-free (-SG) diet (customized from HFK Bioscience) 5 days after tumor injection. For GSK3368715 treatment, mice were treated orally with vehicle (10% DMSO in ddH_2_O) or GSK3368715 at 100 mg/kg every other day. For TAT-peptide treatment, the TAT-methylated peptide or TAT-nonmethylated peptide (10 mg/kg) was injected into the tail vein every other day. Tumor volume was measured every other day using an electronic caliper, and calculated using the formula: Tumor volume (mm^3^) = length (mm) × width^2^ (mm) × 0.52. Mice were euthanized by cervical dislocation at the end of the experiment, after which tumors and major organs were collected by using surgical scissors.

### Immunohistochemical staining

The HCC tissue microarray composed of 42 HCC tissues and paired adjacent normal tissues were obtained from West China Hospital, Chengdu. Patient demographics and clinical characteristics were shown in Supplementary Table 1. IHC staining was performed according to the previous report^72^. Briefly, the paraffin-embedded slides were deparaffinized, rehydrated, and blocked with 3% H_2_O_2_, followed by antigen retrieval in the citrate buffer using a microwave. The sections were then blocked with 10% serum, and subsequently incubated with primary antibodies at 4 °C overnight. The sections were treated with MaxVision HRP-Polymer anti-Mouse/Rabbit IHC Kit (MXB Biotechnology, 5010) and stained with DAB (MXB Biotechnology, 0031). The staining intensity was graded as: negative (0), weak (1), moderate (2), and strong (3). H-Score (0–300) = (1 × % of weak staining) + (2 × % moderate staining) + (3 × % strong staining). The intensities of protein levels were evaluated independently by two experienced investigators.

### Statistics and reproducibility

All experiments were repeated independently with similar results for three times, and all data represent the mean ± SD of three independent experiments unless otherwise specified. GraphPad Prism 8 software was used for statistical analyses. Two-tailed Student’s *t* test was used to compare the variables between two groups. Two-way ANOVA with Bonferroni correction was performed for tumor volume and cell growth rate. Pearson correlation and linear regression were used to determine the correlation in clinical samples. Kaplan–Meier survival curve and the log-rank test were conducted to analyze the survival. All statistical analyses were performed using GraphPad Prism 8.0 software.

### Reporting summary

Further information on research design is available in the Nature Portfolio Reporting Summary linked to this article.

## Supplementary information

Supplementary Information

Description of Additional Supplementary Files

Supplementary Data 1

Reporting Summary

## Data Availability

The raw RNA-seq data generated in this study have been deposited in Gene Expression Omnibus (GEO) under the accession number GSE207435. The proteomics data generated in this study have been deposited in the ProteomeXchange Consortium (http://proteomecentral.proteomexchange.org) via the iProX partner repository with the dataset identifier PXD035287 and PXD035061. The remaining data are available within the Article, Supplementary Information or Source Data file. All relevant data are available upon reasonable request. Source data are provided with this paper.

## Source data

Source Data
