# Supplementary material for: PHGDH arginine methylation by PRMT1 promotes serine synthesis and represents a therapeutic vulnerability in hepatocellular carcinoma
Source: Nat Commun. 2023 Feb 23;14:1011. doi: 10.1038/s41467-023-36708-5 (PMC9950448; doi:10.1038/s41467-023-36708-5)
Supplement: Supplementary file 1 — Supplementary Information [file 41467_2023_36708_MOESM1_ESM.pdf]

Supplementary Information

PHGDH Arginine Methylation by PRMT1 Promotes Serine Synthesis and Represents a Therapeutic Vulnerability in Hepatocellular Carcinoma

Kui Wang, Li Luo, Shuyue Fu, Mao Wang, Zihao Wang, Lixia Dong, Xingyun Wu, Lunzhi Dai, Yong Peng, Guobo Shen, Hai-Ning Chen, Edouard Collins Nice, Xiawei Wei, Canhua Huang

Contents

Supplementary Figures 1-10

Supplementary Tables 1-2

Supplementary Figure 1

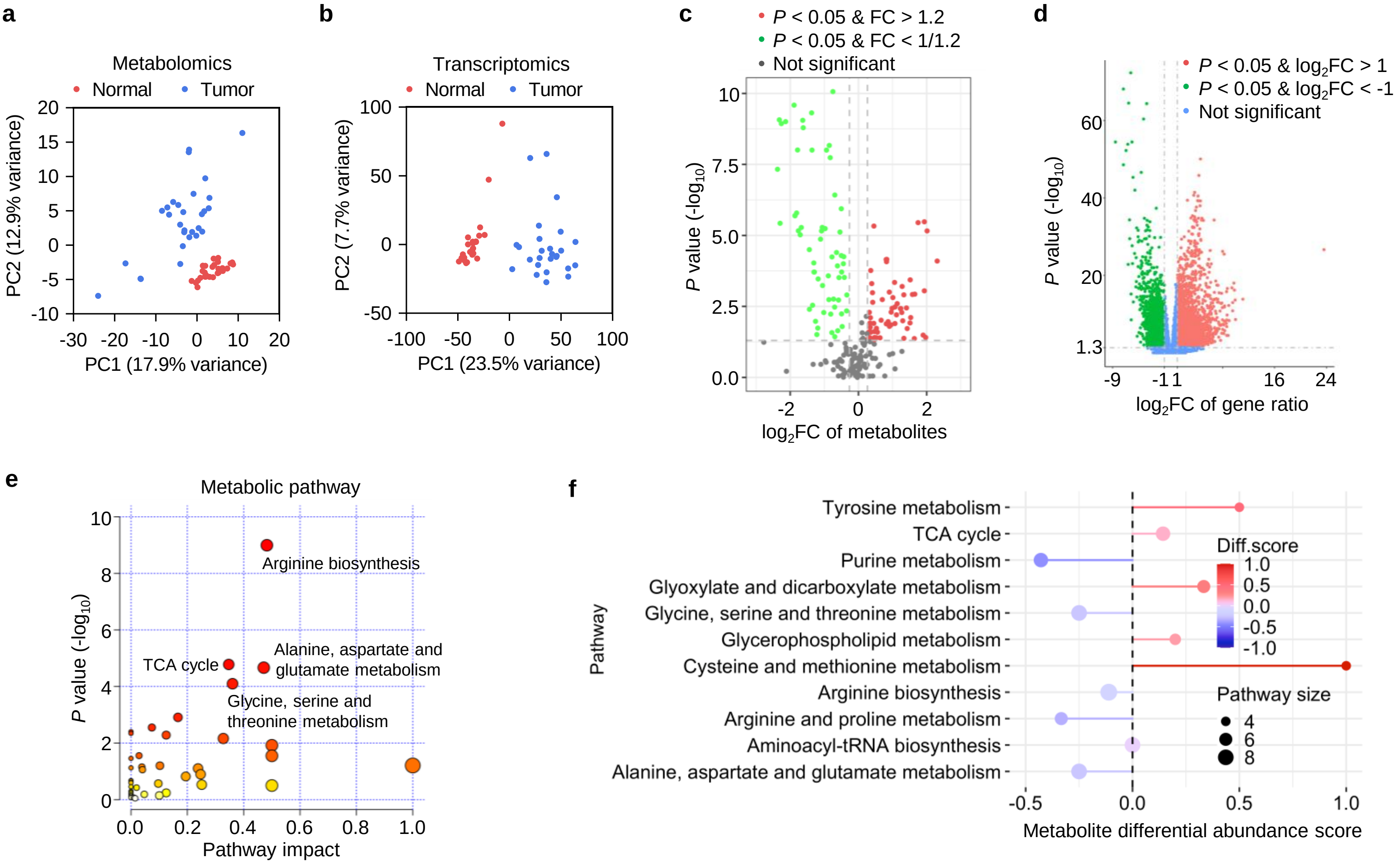

Supplementary Figure 1. Metabolomics and transcriptomics analyses of HCC. a, b Principal component analysis of metabolomics (**a**) and transcriptomics (**b**) data. **c** Volcano plots of differential metabolites in HCC tissues relative to normal tissues (*n* = 29 samples, cohort 1). Statistical analysis was performed using the paired two-tailed Student's t-test. **d** Volcano plots of differential genes in HCC tissues relative to normal tissues (*n* = 27 samples from cohort 1). Statistical analysis was performed using the paired two-tailed Student's t-test with Benjamini-Hochberg correction. **e** Pathway impact of changes in metabolite classes. Statistical analysis was performed using the two-sided hypergeometric test. **f** KEGG pathway-based differential abundance score of differential metabolites in HCC tissues relative to normal tissues. A score of 1 denotes all metabolites in a given pathway increase, while a score of -1 denotes all metabolites in a given pathway decrease. Source data are provided as a Source Data file.

Supplementary Figure 2

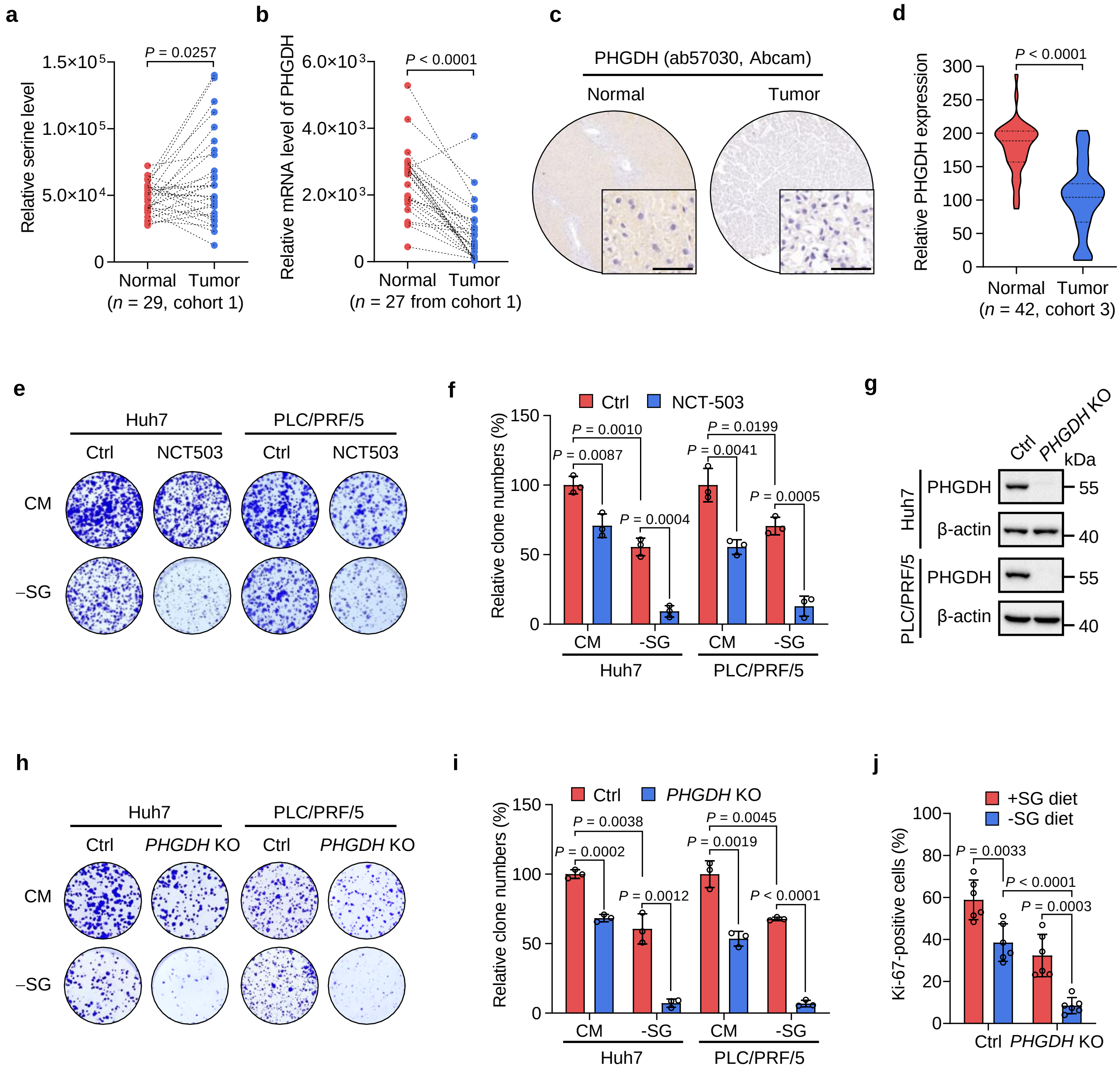

Supplementary Figure 2. PHGDH activity is required for the growth of HCC cells. a Relative serine level obtained from metabolomics analysis of human HCC tissues and paired normal tissues (*n* = 29 samples, cohort 1). Statistical analysis was performed using the paired two-tailed Student's t-test. **b** Relative PHGDH mRNA levels obtained from RNA-seq analysis of human HCC tissues and paired normal tissues (*n* = 27 samples). Statistical analysis was performed using the paired two-tailed Student's t-test. **c, d** Representative images (**c**) and quantitative analysis (**d**) of IHC staining using PHGDH antibody (ab57030, Abcam) in HCC tissues and paired normal tissues (*n* = 42 samples, cohort 3). Scale bars, 50 μm. Statistical analysis was performed using the paired two-tailed Student's t-test. **e, f** Colony formation assay (**e**) and quantification of clone numbers (**f**) of Huh7 and PLC/PRF/5 cells grown in complete (CM) or serine- and glycine-depleted (-SG) medium treated with or without NCT-503 (20 μM). Data are presented as the mean ± SD (*n* = 3 independent experiments). Statistical analysis was performed using the two-tailed Student's *t*-test. **g** Immunoblots for PHGDH in parental (Ctrl) and *PHGDH* KO cells. **h**, **i** Colony formation assay (**h**) and quantification of clone numbers (**i**) of parental (Ctrl) and *PHGDH* KO cells grown in complete (CM) or serine- and glycine-depleted (-SG) medium. Data are presented as the mean ± SD (*n* = 3 independent experiments). Statistical analysis was performed using the two-tailed Student's *t*-test. **j** Huh7 parental and *PHGDH* KO cells were subcutaneously inoculated into nude mice fed with a control (+SG) or serine- and glycine-free diet (-SG diet). Quantitative analysis of IHC staining for Ki-67 in tumor xenografts was shown. Data are presented as the mean ± SD (*n* = 6 mice). Statistical analysis was performed using the two-tailed Student's *t*-test. Source data are provided as a Source Data file.

Supplementary Figure 3

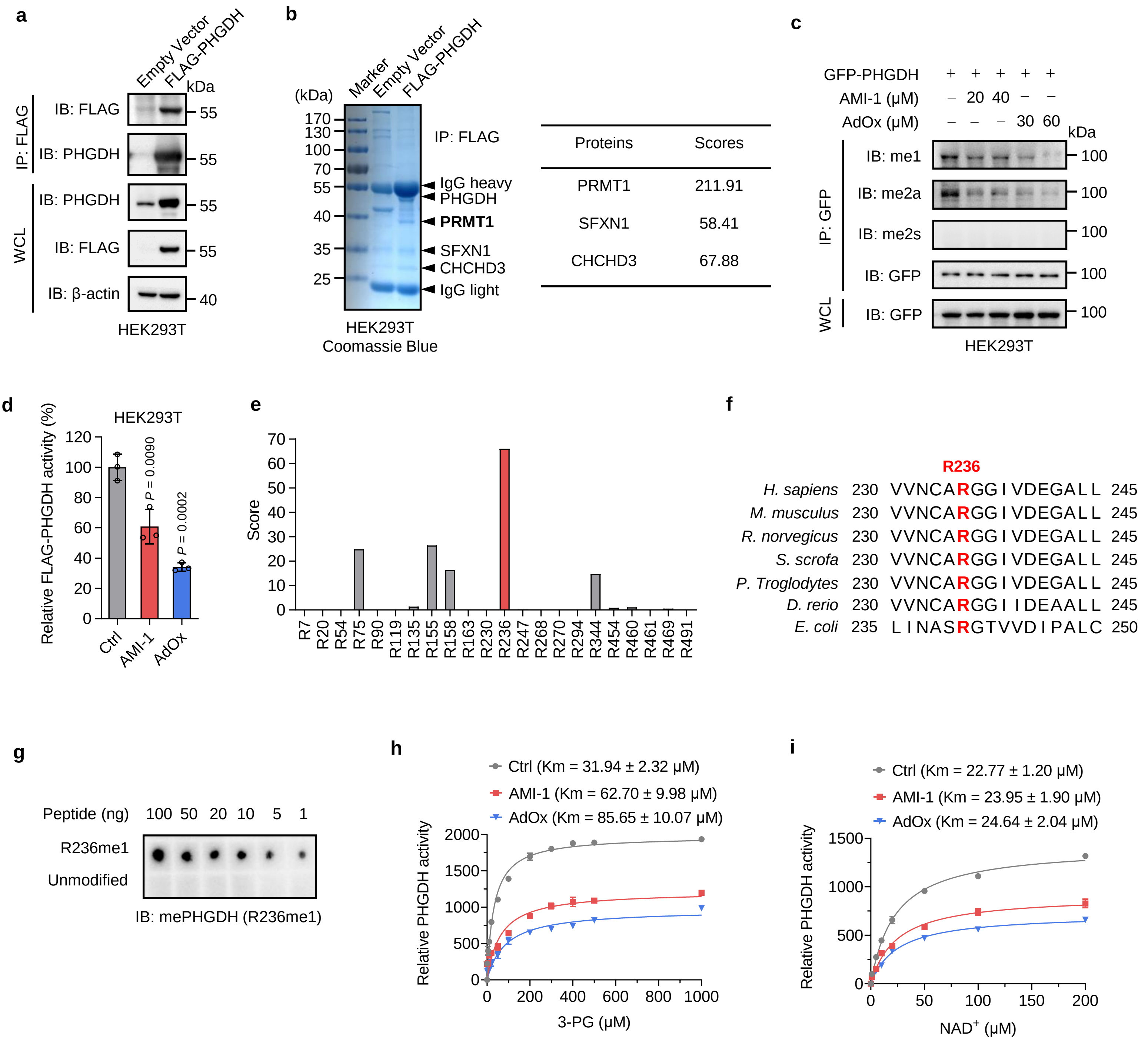

Supplementary Figure 3. PHGDH is methylated at R236, leading to increased catalytic activity. a, b FLAG-PHGDH was immunopurified by FLAG beads in HEK293T cells stably expressing FLAG-PHGDH. The SDS-PAGE gel was immunoblotted with PHGDH and FLAG antibody (**a**), or stained with Coomassie Brilliant Blue (**b**). The protein bands were excised and digested in gel with trypsin followed by LC-MS/MS analysis. **c** GFP-PHGDH was immunoprecipitated in GFP-PHGDH-expressing HEK293T cells treated with AMI-1 or AdOx for 24 h. The methylation level of GFP-PHGDH was determined by immunoblotting using indicated antibodies. **d** FLAG-PHGDH was immunopurified by FLAG beads in HEK293T cells stably expressing FLAG-PHGDH, followed by elution with FLAG peptides. The activity of PHGDH was measured and normalized to FLAG-PHGDH protein. Data are presented as the mean ± SD ($n = 3$ independent experiments), and statistical analysis was performed using the two-tailed Student's *t*-test. **e** Prediction of arginine methylation sites of PHGDH by the GPS-MSP tool. **f** Sequence alignment of PHGDH protein from indicated species. **g** Dot blot analysis of different amounts of R236 mono-methylated (R236me1) peptide or unmodified peptide by a site-specific antibody against R236 mono-methylation (mePHGDH (R236me1)). **h**, **i** The Km value of immunoprecipitated PHGDH for 3-PG (**h**) or NAD^+ (**i**) from cells treated with AMI-1 or AdOx for 24 h. Data are presented as the mean ± SD ($n = 3$ independent experiments). Source data are provided as a Source Data file.

Supplementary Figure 4

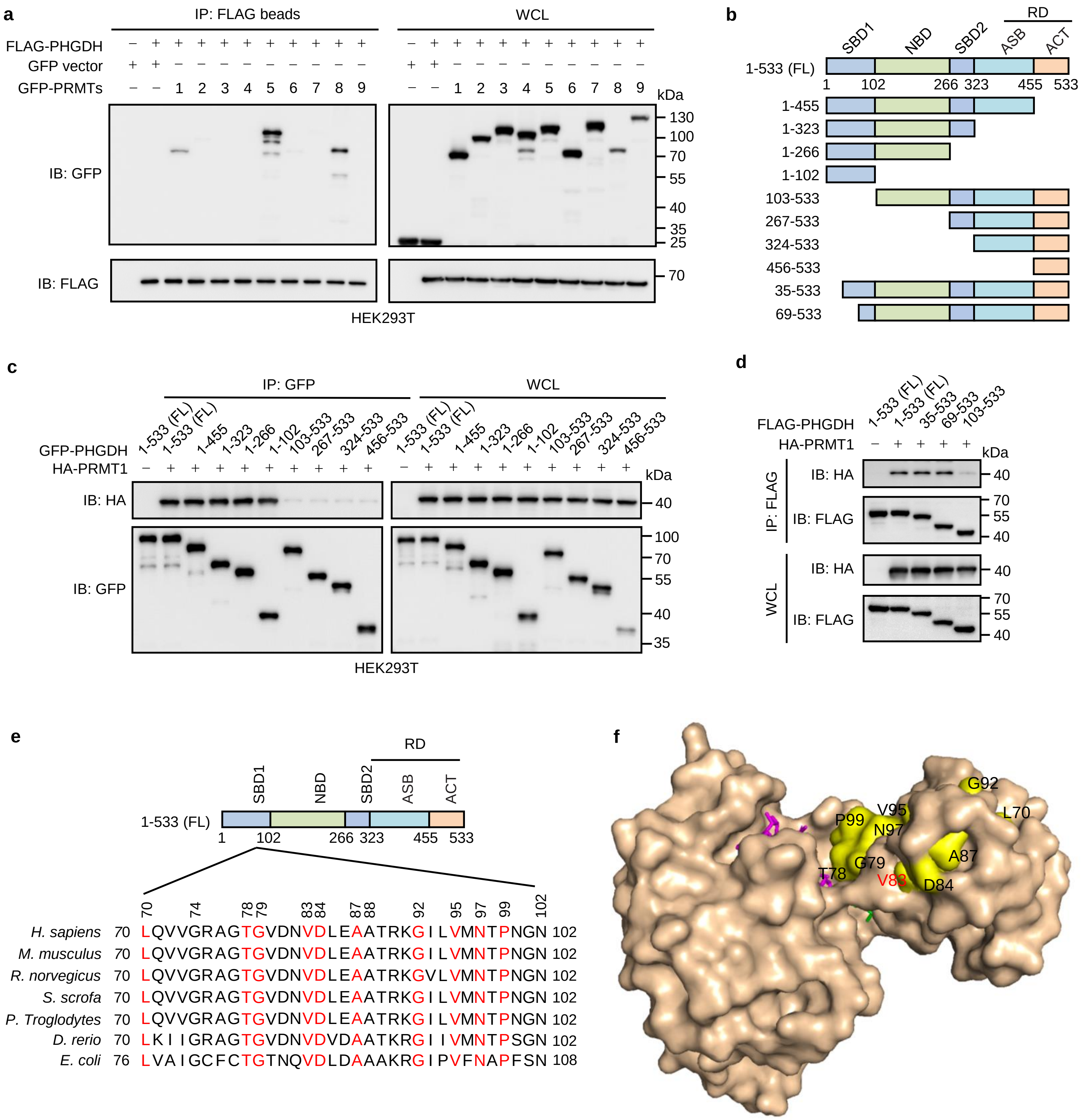

Supplementary Figure 4. V83 in the SBD1 domain of PHGDH is required for PRMT1 binding. **a** FLAG-PHGDH was co-expressed with GFP-tagged PRMTs (PRMT1-9) in HEK293T cells. FLAG-PHGDH was immunopurified with FLAG beads, followed by immunoblotting analysis of GFP-PRMTs using GFP antibody. **b** Schematic representation of full-length (FL) PHGDH and serial truncation mutants. SBD1/2, substrate binding domain 1/2; NBD, nucleotide binding domain; RD, regulatory domain; ASB, allosteric substrate binding; ACT, additional regulatory domain. **c**, **d** GFP-PHGDH FL or indicated truncation mutants were co-expressed with HA-PRMT1 in HEK293T cells. GFP-PHGDH was immunoprecipitated with GFP antibody, followed by immunoblotting analysis of HA-PRMT1 using HA antibody. **e** Amino acids in the 70-102aa region of PHGDH. Red indicates amino acids which were evolutionally conserved and located at the surface of PHGDH protein. **f** Amino acids in the 70-102aa region located at the surface of PHGDH. Results are representative of three independent experiments. Source data are provided as a Source Data file.

Supplementary Figure 5

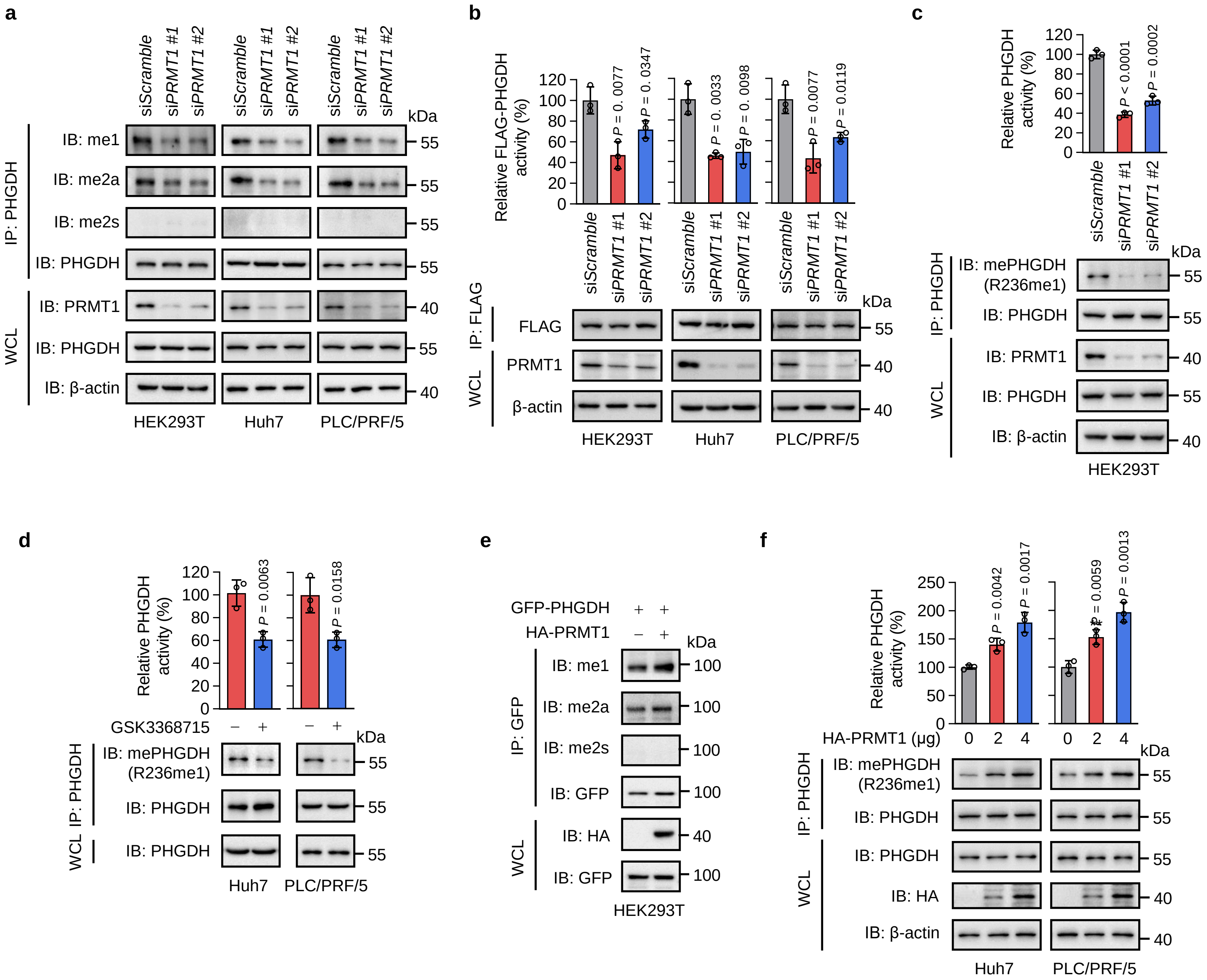

Supplementary Figure 5. PRMT1 promotes PHGDH methylation and activity. a Endogenous PHGDH was immunoprecipitated in cells transfected with si*Scramble* or si*PRMT1*. Immunoblotting was performed with indicated antibodies. **b** FLAG-PHGDH was immunopurified by FLAG beads and eluted by FLAG peptides. PHGDH activity was measured and normalized to FLAG-PHGDH protein. Data are presented as the mean ± SD ($n = 3$ independent experiments), and statistical analysis was performed using the two-tailed Student's *t*-test. **c** Endogenous PHGDH was immunoprecipitated in HEK293T cells transfected with si*Scramble* or si*PRMT1*. Immunoblotting was performed with indicated antibodies. PHGDH activity was measured and normalized to PHGDH protein. Data are presented as the mean ± SD ($n = 3$ independent experiments), and statistical analysis was performed using the two-tailed Student's *t*-test. **d** Endogenous PHGDH was immunoprecipitated in cells treated with or without PRMT1 inhibitor GSK3368715 (2 μM). Immunoblotting was performed with indicated antibodies. PHGDH activity was measured and normalized to PHGDH protein. Data are presented as the mean ± SD ($n = 3$ independent experiments), and statistical analysis was performed using the two-tailed Student's *t*-test. **e** GFP-PHGDH was co-expressed with HA-PRMT1 in HEK293T cells, followed by immunoprecipitation of GFP-PHGDH with GFP antibody. Immunoblotting was performed with indicated antibodies. **f** Endogenous PHGDH was immunoprecipitated in cells transfected with or without HA-PRMT1. Immunoblotting was performed with indicated antibodies. PHGDH activity was measured and normalized to PHGDH protein. Data are presented as the mean ± SD ($n = 3$ independent experiments), and statistical analysis was performed using the two-tailed Student's *t*-test. Source data are provided as a Source Data file.

Supplementary Figure 6

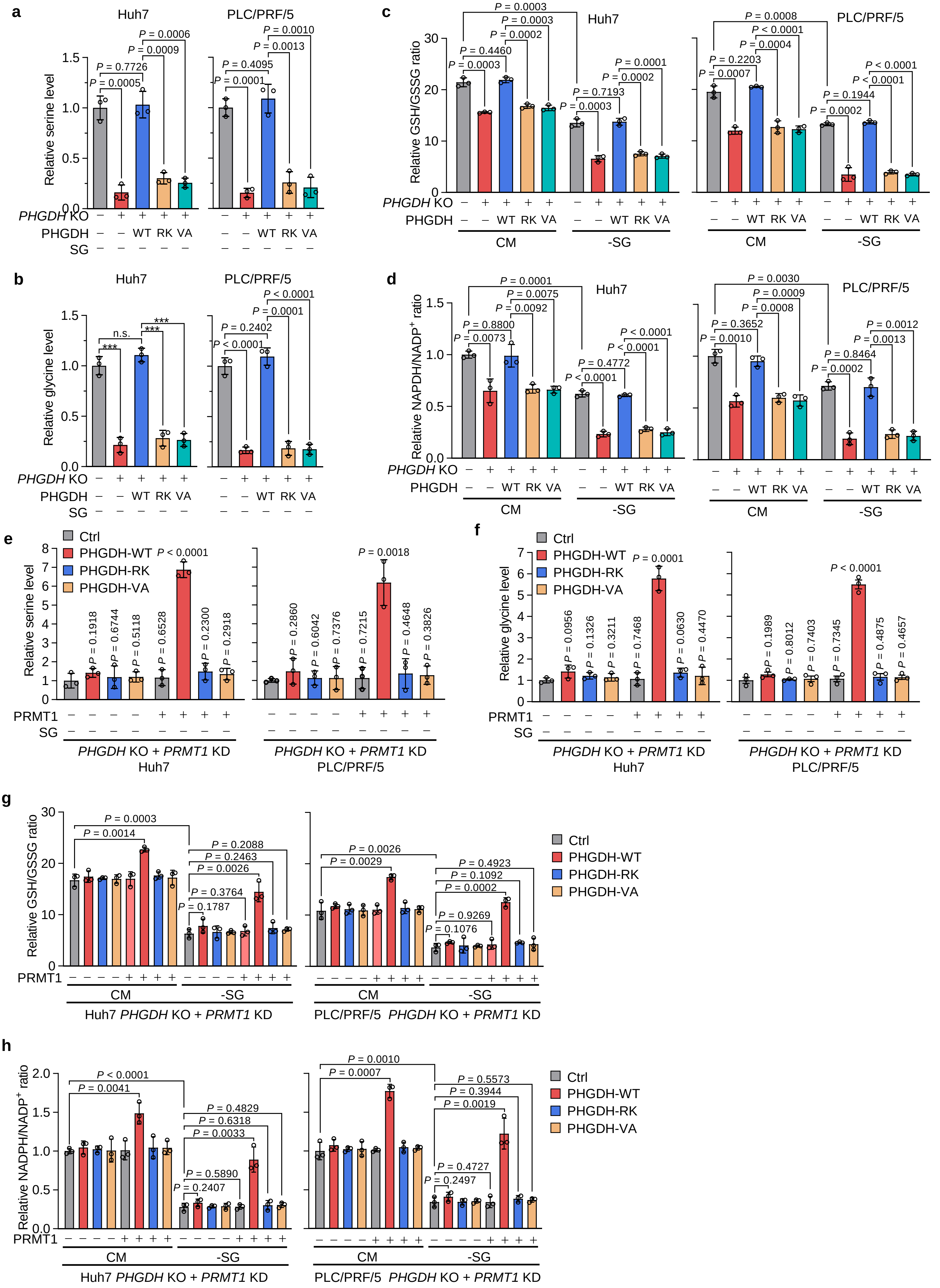

Supplementary Figure 6. R236 methylation of PHGDH mediated by PRMT1 promotes serine synthesis and ameliorates oxidative stress. a-d *PHGDH* was knocked out (*PHGDH* KO) by CRIPSR/Cas9 technology, followed by re-expressing PHGDH WT, R236K (RK) or V83A (VA). Total serine (**a**) and glycine (**b**) levels were measured in these cells grown in -SG medium. GSH/GSSG (**c**) and NADPH/$NADP^+$ ratios (**d**) were determined in these cells grown in CM or -SG medium. Data are presented as the mean ± SD ($n = 3$ independent experiments), and statistical analysis was performed using the two-tailed Student's *t*-test. **e-h** *PHGDH* KO plus *PRMT1* KD cells were rescued with PHGDH WT, R236K (RK) or V83A (VA), combining with or without PRMT1 re-expression. Total serine (**e**) and glycine (**f**) levels were measured in cells grown in -SG medium. GSH/GSSG (**g**) and NADPH/$NADP^+$ ratios (**h**) were determined in cells grown in CM or -SG medium. Data are presented as the mean ± SD ($n = 3$ independent experiments), and statistical analysis was performed using the two-tailed Student's *t*-test. Source data are provided as a Source Data file.

Supplementary Figure 7

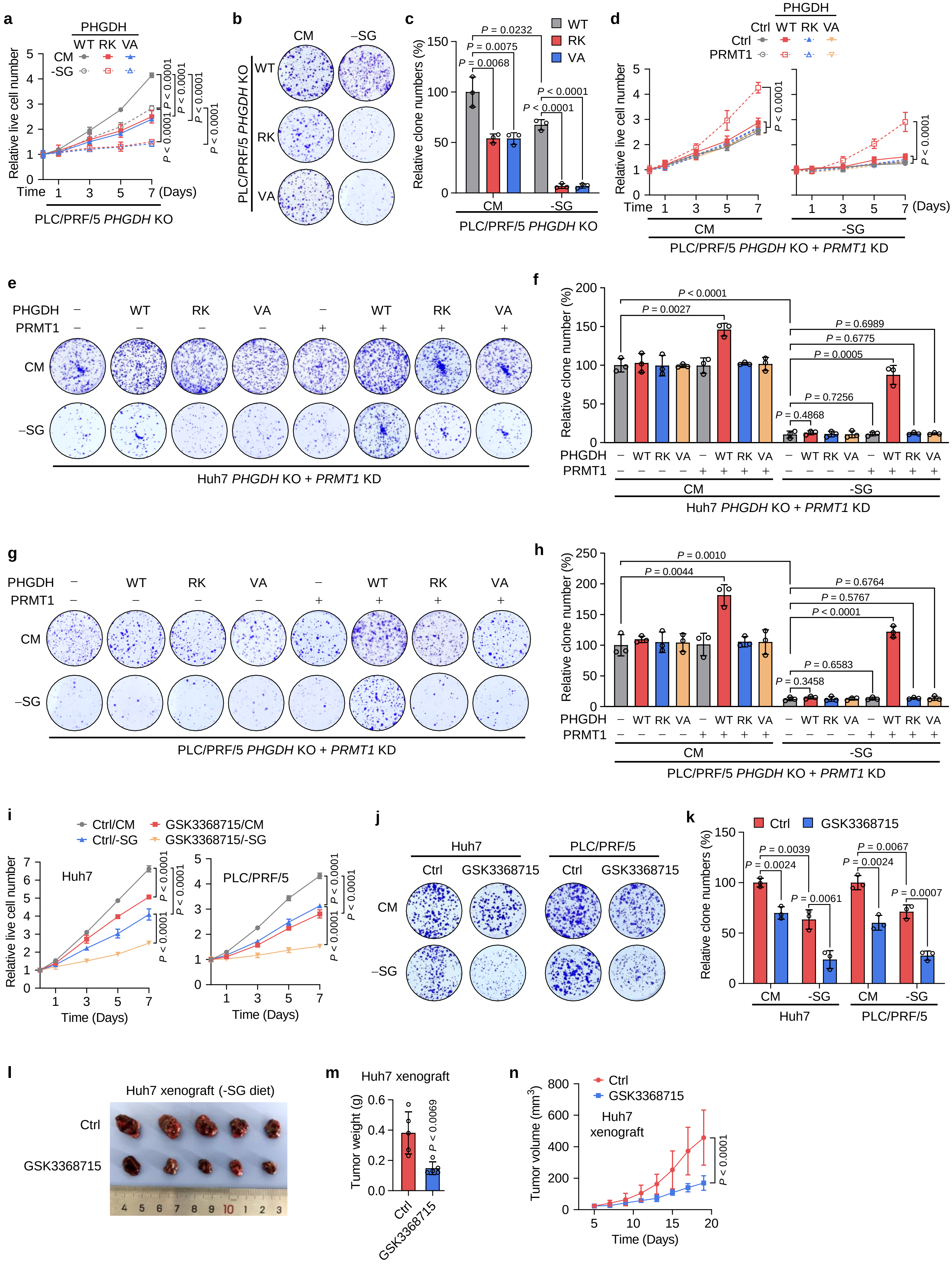

Supplementary Figure 7. R236 methylation of PHGDH mediated by PRMT1 promotes the growth of HCC cells. a-c *PHGDH* KO PLC/PRF/5 cells were re-expressed with PHGDH WT, R236K (RK) or V83A (VA). Growth rates (**a**), colony formation assay (**b**), and quantification of clone numbers (**c**) of these cells grown in CM or -SG medium. Data in **a** are presented as the mean ± SD ($n = 5$ independent experiments), and statistical analysis was performed using the two-way ANOVA with Bonferroni correction. Data in **c** are presented as the mean ± SD ($n = 3$ independent experiments), and statistical analysis was performed using the two-tailed Student's *t*-test. **d-h** *PHGDH* KO plus *PRMT1* KD cells were rescued with PHGDH WT, R236K (RK) or V83A (VA), combining with or without PRMT1 re-expression. Growth rates (**d**), colony formation assay (**e**, **g**), and quantification of clone numbers (**f**, **h**) of these cells grown in CM or -SG medium. Data in **d** are presented as the mean ± SD ($n = 5$ independent experiments), and statistical analysis was performed using the two-way ANOVA with Bonferroni correction. Data in **f** and **h** are presented as the mean ± SD ($n = 3$ independent experiments), and statistical analysis was performed using the two-tailed Student's *t*-test. **i-k** Growth rates (**i**), colony formation assay (**j**) and quantification of clone numbers (**k**) of Huh7 and PLC/PRF/5 cells grown in CM or -SG medium treated with or without GSK3368715 (2 μM). Data in **i** are presented as the mean ± SD ($n = 4$ independent experiments), and statistical analysis was performed using the two-way ANOVA with Bonferroni correction. Data in **k** are presented as the mean ± SD ($n = 3$ independent experiments), and statistical analysis was performed using the two-tailed Student's *t*-test. **l-n** Images (**l**), weight (**m**), and volume (**n**) of Huh7 tumor xenografts from mice fed with a -SG diet and treated with or without GSK3368715. Data are presented as the mean ± SD (n = 5 mice). Statistical analysis in **m** was performed using the two-tailed Student's *t*-test, and statistical analysis in **n** was performed using the two-way ANOVA with Bonferroni correction. Source data are provided as a Source Data file.

Supplementary Figure 8

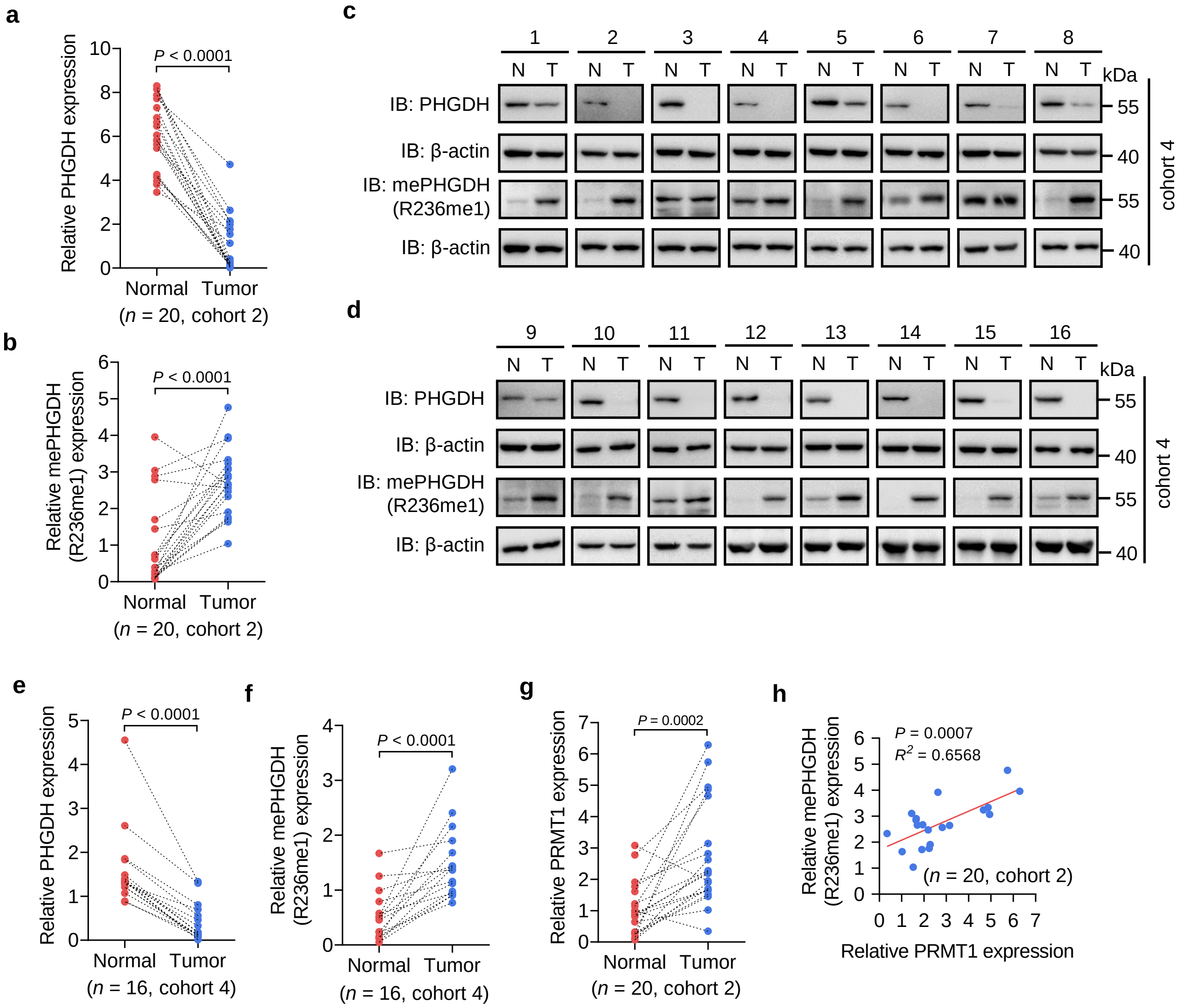

Supplementary Figure 8. PHGDH methylation level is elevated and positively correlated with PRMT1 protein level in human HCC tissues. a-b Quantitation of the levels of PHGDH (**a**) and mePHGDH (R236me1) (**b**) in HCC tissues and paired normal tissues based on band intensity shown in Figs. 6e, f (*n* = 20 samples, cohort 2). Statistical analysis was performed using the paired two-tailed Student's *t*-test. **c, d** Immunoblotting analysis with PHGDH and mePHGDH (R236me1) antibody in 16 HCC tissues (T) and paired normal tissues (N) (*n* = 16, cohort 4). **e-f** Quantitation of the levels of PHGDH (**e**) and mePHGDH (R236me1) (**f**) in HCC tissues and paired normal tissues based on band intensity shown in **c, d** (*n* = 16 samples, cohort 4). Statistical analysis was performed using the paired two-tailed Student's *t*-test. **g** Quantitation of the levels of PRMT1 in HCC tissues and paired normal tissues based on band intensity shown in Figs. 6e, f (*n* = 20 samples, cohort 2). Statistical analysis was performed using the paired two-tailed Student's *t*-test. **h** Pearson correlation test analyzing the relationship between the IHC staining intensity of mePHGDH (R236me1) and PRMT1 in HCC tissues (*n* = 20 samples, cohort 2). Source data are provided as a Source Data file.

Supplementary Figure 9

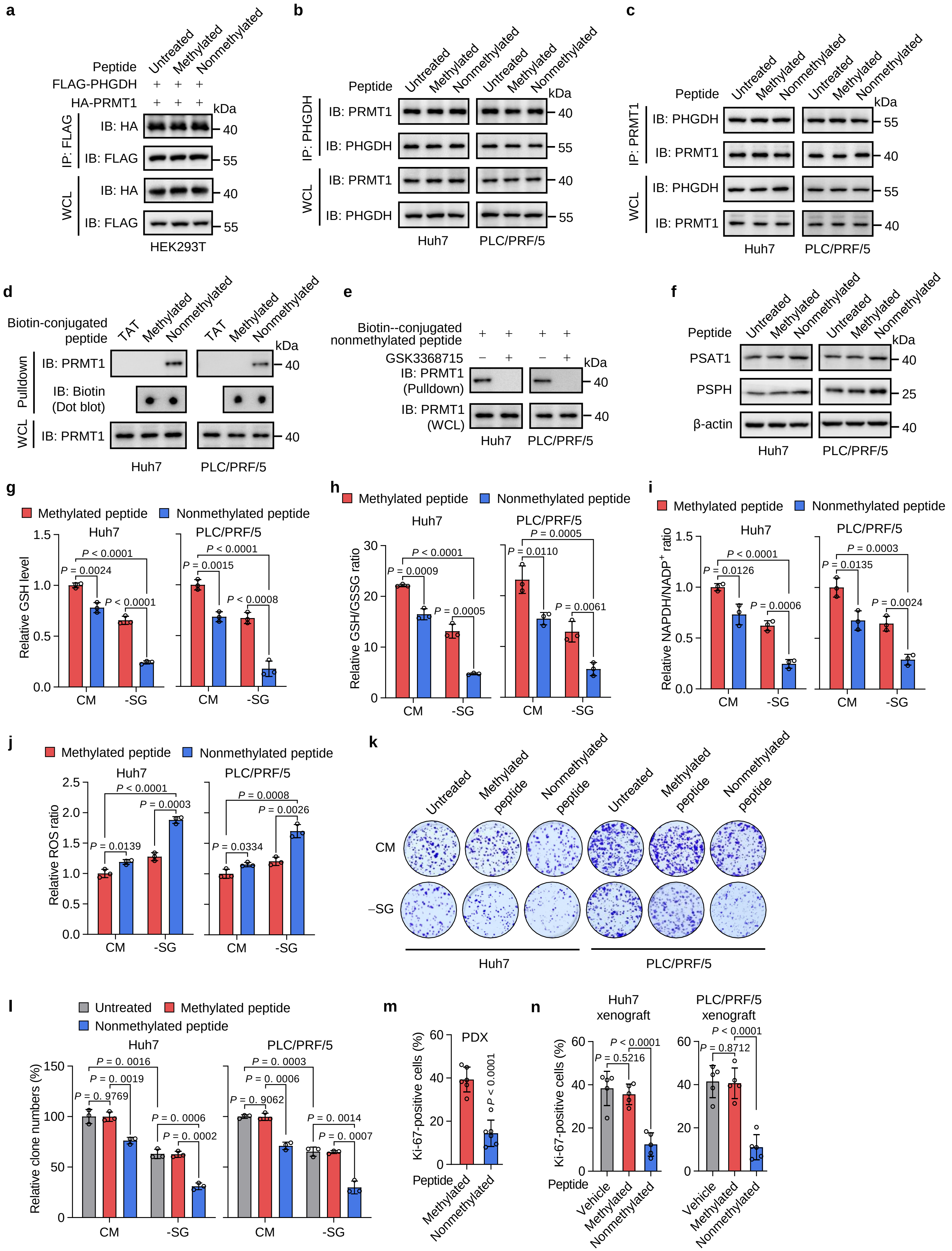

Supplementary Figure 9. Inhibition of PHGDH methylation using a synthesized nonmethylated peptide restrains HCC growth. a FLAG-PHGDH was co-expressed with HA-PRMT1 in HEK293T cells followed by treatment with the methylated or nonmethylated peptides (20 μM) for 24 h. FLAG-PHGDH was immunopurified by FLAG beads. Immunoblotting was performed with indicated antibodies. **b, c** Reciprocal co-IP analysis of PHGDH and PRMT1 in Huh7 and PLC/PRF/5 cells treated with the methylated or nonmethylated peptides (20 μM) for 24 h. **d** Huh7 and PLC/PRF/5 cells treated with the biotin-conjugated TAT, methylated, or nonmethylated peptides (20 μM) for 24 h were lysed and subjected to immunoprecipitation by streptavidin beads. Immunoblotting was performed with PRMT1 antibody. Dot blot of immunoprecipitated biotinylated peptides was performed using biotin antibody. **e** Huh7 and PLC/PRF/5 cells were pretreated with GSK3368715 for 6 h, and then treated with the biotinylated nonmethylated peptides (20 μM) for 24 h. The cell lysates were immunoprecipitated by streptavidin beads, followed by immunoblotting using PRMT1 antibody. **f** Immunoblots for PSAT1 and PSPH in Huh7 and PLC/PRF/5 cells treated with the methylated or nonmethylated peptides (20 μM) for 24 h. **g-j** GSH level (**g**), GSH/GSSG ratio (**h**), NADPH/$NADP^+$ ratio (**i**), and ROS levels (**j**) in cells grown in CM or -SG medium with treatment of the methylated or nonmethylated peptides (20 μM) for 24 h. Data are presented as the mean ± SD ($n = 3$ independent experiments). Statistical analysis was performed using the two-tailed Student's *t*-test. **k-l** Colony formation assay (**k**) and quantification of clone numbers (**l**) of cells grown in CM or -SG medium with treatment of the methylated or nonmethylated peptides (20 μM) for 24 h. Data are presented as the mean ± SD ($n = 3$ independent experiments). Statistical analysis was performed using the two-tailed Student's *t*-test. **m**, **n** Quantitative analysis of IHC staining for Ki-67 in PDX tumors (**m**), and Huh7 and PLC/PRF/5 tumor xenografts (**n**). Data are presented as the mean ± SD ($n = 6$ mice in **m**, and $n = 5$ mice in **n**). Statistical analysis was performed using the two-tailed Student's *t*-test. Source data are provided as a Source Data file.

Supplementary Figure 10

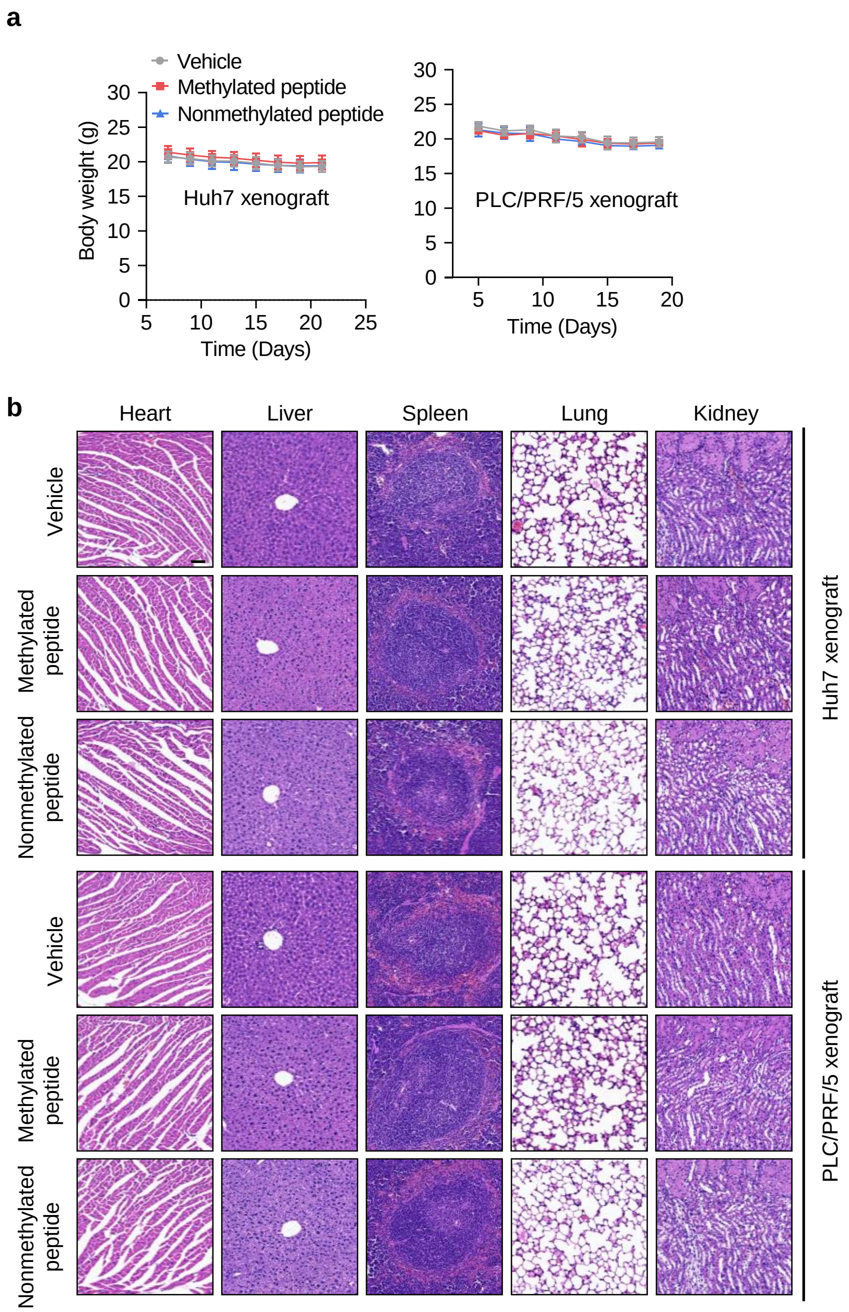

Supplementary Figure 10. The synthesized peptide has no obvious toxic effects in mice. a Body weight changes of mice bearing Huh7 and PLC/PRF/5 tumor xenografts. Data are presented as mean ± SD (*n* = 5 mice). **b** H&E staining of major organs from Huh7 and PLC/PRF/5 tumor-bearing mice fed with a -SG diet and treated with the methylated or nonmethylated peptides. Scale bars, 50 µm. Source data are provided as a Source Data file.

Supplementary Table 1. Clinical characteristics of HCC patients.

Characteristics	HCC (*n* = 29, cohort 1)	HCC (*n* = 20, cohort 2)	HCC (*n* = 42, cohort 3)	HCC (*n* = 16, cohort 4)
Age, year				
Median (range)	57 (36-73)	58 (38-71)	54.5 (36-76)	53 (34-73)
Gender, *n* (%)				
Female	14 (48.3)	4 (20.0)	10 (23.8)	5 (31.3)
Male	15 (51.7)	16 (80.0)	32 (76.2)	11 (68.7)
Tumor size, cm (%)				
< 5	17 (58.6)	11 (55.0)	12 (28.6)	7 (43.8)
≥ 5	12 (17.4)	9 (45.0)	30 (71.4)	9 (56.2)
TNM stage, *n* (%)				
I	12 (41.4)	12 (60.0)	8 (19.0)	4 (25.0)
II	12 (41.4)	6 (30.0)	27 (64.3)	8 (50.0)
III	5 (17.2)	2 (10.0)	7 (16.7)	4 (25.0)

Supplementary Table 2. Gender-based analysis comparing the relative mePHGDH (R236me1), PRMT1, or serine level in HCC tissues from patients with different genders.

	HCC cohort 1 (14 females and 15 males)	HCC cohort 2 (4 females and 16 males)	HCC cohort 3 (10 females and 32 males)	HCC cohort 4 (5 females and 11 males)
Relative mePHGDH (R236me1) expression	/	$P = 0.7120$	$P = 0.2172$	$P = 0.5135$
Relative PRMT1 expression	/	$P = 0.1413$	$P = 0.9712$	/
Relative serine level	$P = 0.3065$	$P = 0.4447$	/	/

P values were derived from the two-tailed Student's t-test ($n = 29$, cohort 1; $n = 20$, cohort 2; $n = 42$, cohort 3; $n = 16$, cohort 4).
