# Supplementary material for: PHGDH arginine methylation by PRMT1 promotes serine synthesis and represents a therapeutic vulnerability in hepatocellular carcinoma
Source: Nat Commun. 2023 Feb 23;14:1011. doi: 10.1038/s41467-023-36708-5 (PMC9950448; doi:10.1038/s41467-023-36708-5)
Supplement: Supplementary file 2 — Description of Additional Supplementary Files [file 41467_2023_36708_MOESM2_ESM.pdf]

Description of Additional Supplementary Files

File Name: Supplementary Data 1
Description: Metabolites with differential abundance in HCC tissues compared with adjacent normal tissues.
